# *Nigella sativa* L. Phytochemistry and Pharmacological Activities: A Review (2019–2021)

**DOI:** 10.3390/biom12010020

**Published:** 2021-12-23

**Authors:** Mohammed Dalli, Oussama Bekkouch, Salah-eddine Azizi, Ali Azghar, Nadia Gseyra, Bonglee Kim

**Affiliations:** 1 Laboratory of Bioresources, Biotechnology, Ethnopharmacology and Health, Faculty of Sciences, University Mohammed the First, P.O. Box 524, 60000 Oujda, Morocco; oussamabekkouch@hotmail.fr (O.B.); azizi.salah-eddine@ump.ac.ma (S.-e.A.); a.azghar@ump.ac.ma (A.A.); ngseyra@hotmail.com (N.G.); 2 Department of Pathology, College of Korean Medicine, Kyung Hee University, Seoul 02447, Korea; 3 Korean Medicine-Based Drug Repositioning Cancer Research Center, College of Korean Medicine, Kyung Hee University, Seoul 02447, Korea

**Keywords:** *Nigella sativa*, thymoquinone, oil, organic extracts, pharmacological properties

## Abstract

Medicinal and aromatic plants are mainly characterized by the presence of different bioactive compounds which exhibit various therapeutic activities. In order to investigate the different pharmacological properties of different *Nigella sativa* extracts, a multitude of research articles published in the period between 2019 and 2021 were obtained from different databases (Scopus, Science Direct, PubMed, and Web of Science), and then explored and analyzed. The analysis of the collected articles allows us to classify the phytochemicals and the pharmacological activities through their underlying molecular mechanisms, as well as to explore the pharmacological activities exhibited by several identified compounds in *Nigella sativa* which allow a better understanding, and better elucidation, of the bioactive compounds responsible for the pharmacological effects. Also shown are the existence of other bioactive compounds that are still unexplored and could be of great interest. This review could be taken as a guide for future studies in the field.

## 1. Introduction

In emerging countries generally and Morocco particularly, the population have used medicinal plants for primary health care due to their low cost and availability [1]. *Nigella sativa* (*NS*), a Ranunculaceae commonly known as “black cumin” or “black seeds”, is an annual plant that is widely distributed, particularly in North Africa, Middle East, Europe, and Asia [2].

The black cumin has traditionally been used in Arab countries, the Indian subcontinent, and Europe for millennia for culinary and medicinal purposes [3]. The *NS* is an annual herbaceous hermaphrodite plant with a height of around 60 cm. The plant is characterized by branched erect stems, that become empty with age with a green to dark green color. It’s leaves have a green color that turns to red with age. The *NS* lower flowers are short and petaloid while the upper ones are long. The plant flowering begins in April until August, mainly formed by five petals with a diameter of 20 to 35 mm. Flowers are characterized by a green color at a young age which turn to blue in maturity. The plant’s fruits are composed of three to six carpels, and each one of them contains seeds. The seeds are of ovoid size (2 to 3.5 mm) composed of three to four finely granulated angles, their color becomes black after maturity and exposure to the air [4]. The black cumin is a natural remedy for many pathologies, especially for the treatment of asthma, inflammation, cough, eczema, and flu-like conditions [5]. The seeds are used as a diuretic, carminative, and dewormer [5]. According to a belief commonly accepted among Muslims, “Habba sawda” as it is called in Arabic, is a panacea used to treat all ailments except to prevent from death [5]. In Moroccan traditional medicine, the plant is used to treat illnesses such as allergy, heart disease, hypertension, scarring, dermatitis, abdominal pain, stomach ache, vomiting, osteoarthritis, and rheumatic pain [6]. The black cumin is mainly formed by non-volatile compounds [7], such as flavonoids, phenolic acids, tannins, and a volatile part containing terpene compounds [8]. Various extracts and other bioactive compounds derived from the seeds, particularly the essential oil and its major compound, thymoquinone (TQ), are responsible for various biological activities, particularly antioxidant, anti-inflammatory, antihepatotoxic, analgesic, antineoplastic, antimutagenic, anti-nephrotoxic immunostimulant, hypoglycemic, antiulcer, antimicrobial and antiparasitic activities [5,9]. The *NS* seeds have been mentioned to have low toxicity [3]. Several review articles have been recently published on the different effects of *NS*. A review assessed by Hwang et al. reported scientific evidence on the use of the *NS* extract in the treatment of dermatological disorders [10]. While the reviews conducted by Alhmied et al., and Hossain et al. focused on the anticancer and the molecular mechanisms of the TQ by the inhibition of the cell proliferation, and the microbial pharmacology of this bioactive compound respectively [11,12]. While another review assessed the TQ’s therapeutic potential against the progression of atherosclerosis and its associated complications [13]. While another review summarizes the possible immune system boosting effect and the different phytochemicals that could influence and reinforce the immune response [14]. The nutritional value of the *NS* and its bioactive compound has been mentioned as a protective agent against food poisoning [15], and the pharmacology and safety of the plant used traditionally on a large scale by the population [16]. The present study aims to highlight the research trends on the *Nigella sativa* by summarizing the pharmacological activities and the described molecular mechanisms involved. This study also allows for the summarizing of bioactive molecules other than TQ that have shown a very strong pharmacological potential. Furthermore, this review sheds light on some molecules, such as alkaloids, that are identified but still not explored enough pharmacologically. Furthermore, our review was assessed for a better direction of future research studies for a potential application in order to improve health conditions. 

## 2. Methodology of Research

This article is a comprehensive literature review that was assessed to provide an overview of the phytochemistry and pharmacological activities of *NS*. Our literature search was based on Scopus, PubMed, ScienceDirect, and Web of Science. The followed keywords were, *Nigella sativa*, black cumin, phytochemistry, pharmacology. Concerning the pharmacological activities several keywords were used for each activity “antioxidant, antimicrobial, anticancer, anti-inflammatory, immunomodulatory, cardioprotective, antihypertensive, antidiabetic, lipid profile”. The bibliographic results were filtered and examined. All research articles published during three years starting from 2019 till 2021 were the subjects of our study. 

## 3. *Nigella sativa* Phytochemistry

### 3.1. Volatile Compounds

**Figure 1:**
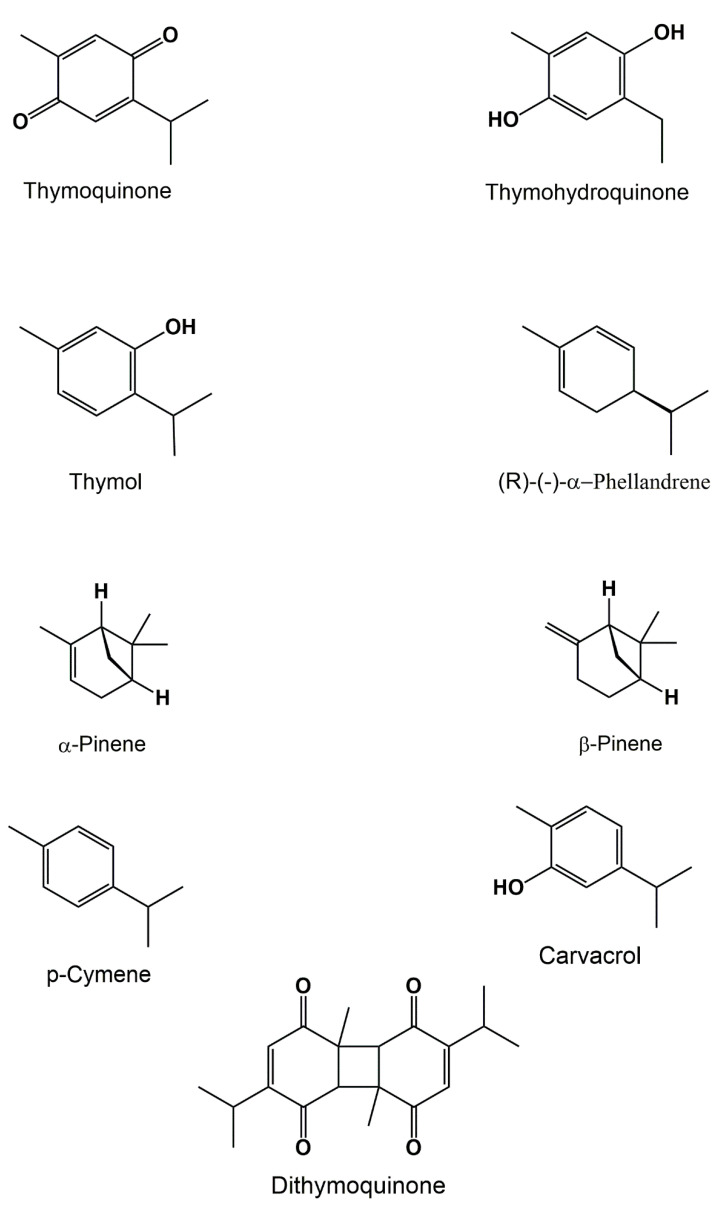
Some of the chemical compounds identified in the black cumin seeds using the GC-MS.

Different studies assessed on the *NS* essential oil have revealed the presence of different molecules of different nature (Figure 1). They include mono-terpene, di-terpene, sesquiterpenes, monoterpenoid alcohols, and ketone. Among them, we cite thymoquinone (TQ), thymohydroquinone, thymol, carvacrol, Phellandrene, α-pinene, and β-pinene [8,17]. 

### 3.2. Phenolic Acids and Flavonoids

**Figure 2:**
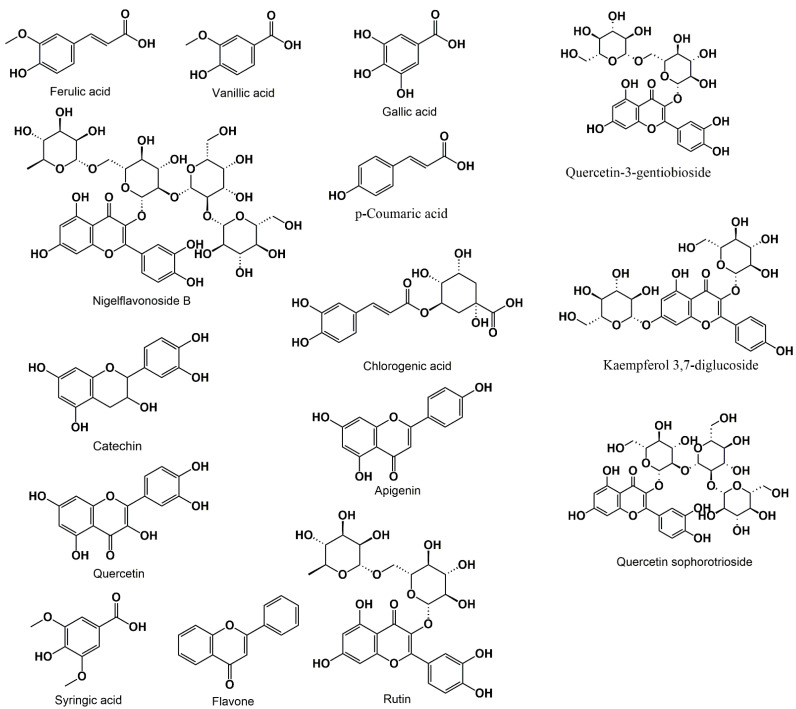
Phenolic compounds present in *Nigella sativa* L.

All these flavonoids exhibit antioxidant properties and allow the protection of the organism against free radicals. Also, flavonoids cause a decrease in inflammatory cell adhesion to the subendothelium and therefore induce a decrease in the inflammatory response. Inhibition of peroxidase activity is another benefit of flavonoids, which allows the prevention from oxygen reactive species [18]. Among the extracted phenolic compounds from the *NS* seeds (Figure 2), we mention gallic acid, ferulic acid, vanillic acid, p-coumaric acid, chlorogenic acid, catechin, quercetin, apigenin, rutin, nigelflavonoside B, and flavone [19,20]. 

### 3.3. Alkaloids

**Figure 3:**
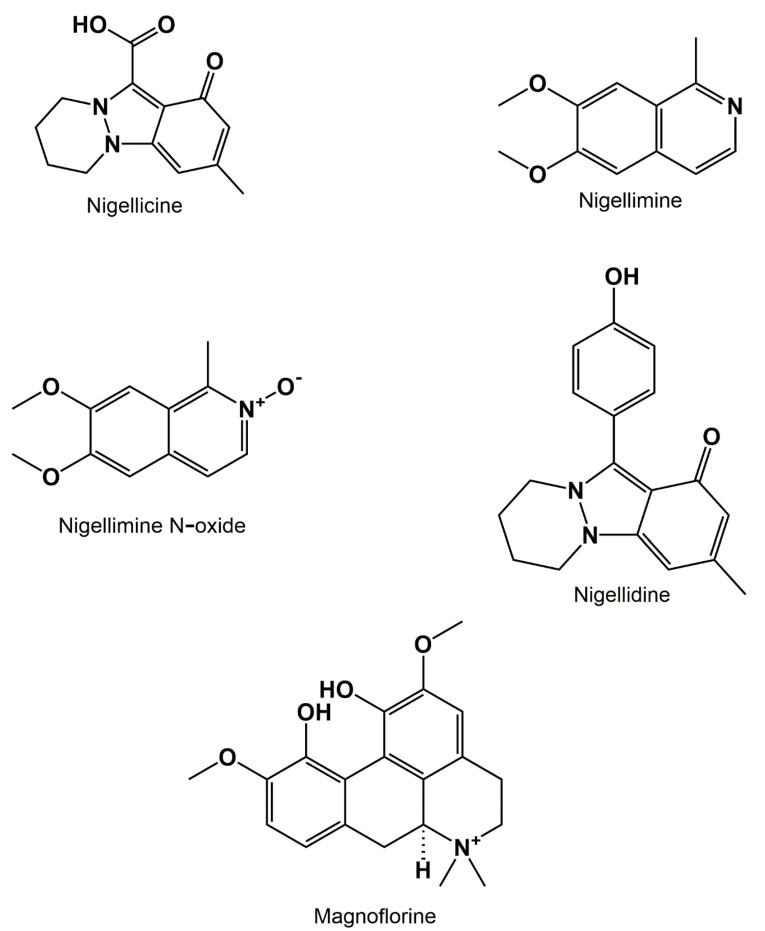
*Nigella sativa* isolated and identified alkaloids.

Between the years 1985 and 1995 different *NS* alkaloids were isolated and identified (Figure 3) such as nigellicine which is composed of an indazole nucleus [21], nigellimine which is an isoquinoline molecule [22], also nigellimine N-oxide, and finally nigellidine which is a molecule of indazole nature [23]. While another molecule called magnoflorine was recently identified in the aerial part of the NS plant [20].

### 3.4. Saponins

Saponins are part of the secondary metabolites present in black cumin, their structures contain steroids or aglycone triterpenes that are linked by a glycosidic bond to one or more oligosaccharides. The presence of polar groups (carbohydrates) and non-polar groups (steroids and triterpenes) give saponins a great capacity to bind to the cell surface and their membrane components [24]. In a study on the black cumin methanolic extract, several saponins were identified [25];

3-O-α-L-rha-(1-2)α-L-ara-28-O-α-Lrha(1-4)-β-D-glu(1-6)-β-D-glu-hederagenine;

3-O-β-D-xyl(1-3)-α-L-rha-(1-2)-α-L-ara-hederagenine. 

**Figure 4:**
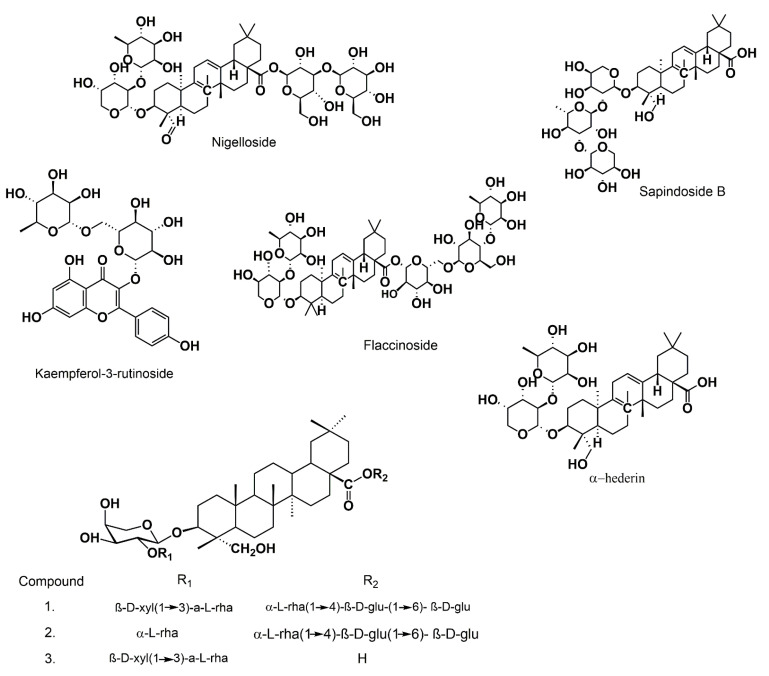
Some saponins isolated from the aerial part of *NS*.

In another study several saponins were isolated and identified in the aerial part of the plant [20], among these we cite, nigelloside, *Kaempferol 3-O-rutinoside* and Flaccidoside (Figure 4).

### 3.5. Fatty Acids

**Figure 5:**
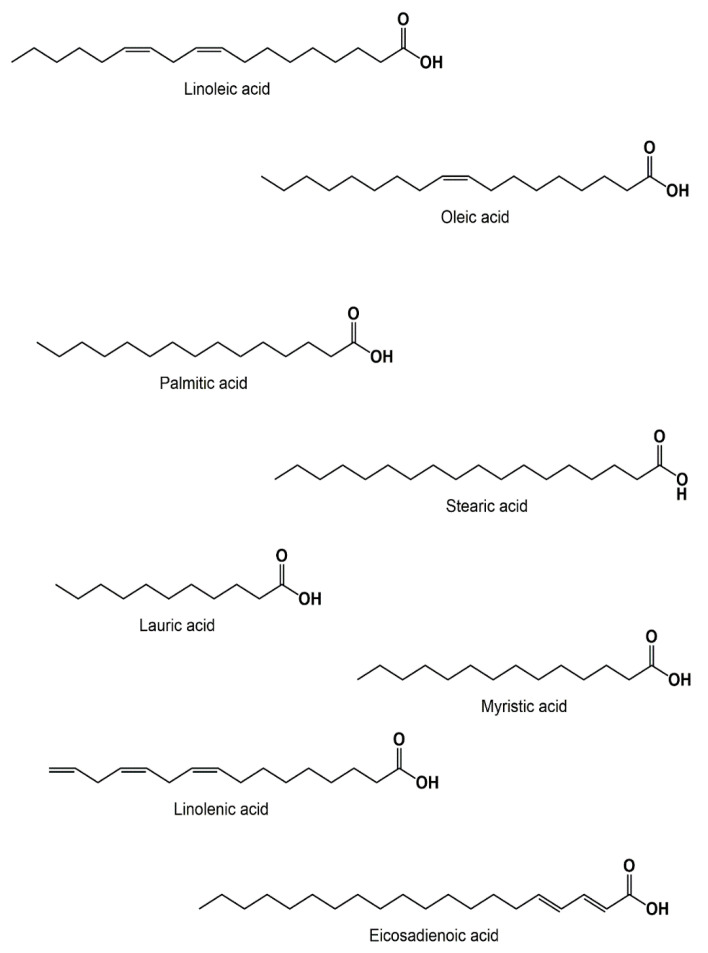
Examples of fatty acids found in *NS* seeds.

The black cumin seeds were found to contain crude fiber, minerals (Na, Cu, Zn, P, and Ca), and vitamins such as thiamine, niacin and folic acid [26]. Furthermore, the *NS* possesses different types of fatty acids that were determined using the GC-MS technique [19,27] (Figure 5). The linoleic acid (55.6%) was among the most abundant fatty acids present in the *NS* seeds, followed by oleic acid (23.4%), and palmitic acid (12.5%). While, stearic acid, lauric acid, myristic acid, linolenic acid, and Eicosadienoic acid are present in small amounts with a percentage ranging from 0.5% to 3.4% [28].

## 4. Pharmacological Properties of *Nigella sativa*

### 4.1. Antioxidant Activity

#### 4.1.1. In Vitro

A study carried out by Mahmoud et al., 2021 [29] demonstrated a high antiradical scavenging activity of *NS* seeds which was attributed to the high content of phenolic and flavonoid compounds. A study by Bocsan et al., 2021 [30], indicated that the *NS* oil has a high antiradical scavenging activity when tested against 2,2-diphenyl-1-picrylhydrazyl (DPPH) radical. Another in vitro study based on the exposition of washed erythrocytes to H_2_O_2_ (10 mM) in the presence and the absence of methanolic extract of *NS* indicated that the preincubation of the washed erythrocytes with methanolic extract at different concentrations showed a decrease of the malondialdehyde (MDA) and antioxidant enzymes, and an increase of the glutathione GSH in a dose-dependent manner. In addition to the mentioned effects, anti-hemolytic activity has also been noticed [31] (Table 1). These results are in accordance with those obtained in the study of Farshori et al., 2021 [32] where the pretreatment of human umbilical vein endothelial cells (HUVEC) with different doses of the ethanolic extract of the *NS* seeds protected the cells from the negative effect of the H_2_O_2_ which confirms the antioxidant effect of the plant. The radiations have an enormous interactive capacity with human tissue and can cause free radical generation by radiolysis of water molecules which induce damage at the level of the DNA and cell death. The irradiation of Keratinocyte cells by the ultraviolet A-rays (UVA) (320 and 400 nm) can cause oxidative stress, inflammation, and apoptosis. So, the pretreatment of HaCaT by the TQ induced a protective effect against the UVA light by inhibition of the cyclooxygenase-2 (COX-2), and this by the activation of the NrF2/ARE pathway. The COX-2 has already been mentioned to be an indicator of epithelial cell proliferation, oxidative stress, and inflammation. Hence, the TQ can attenuate the oxidative stress, and inflammatory activity at the keratinocytes level [33]. The investigation of the combination between the aqueous extract and honey showed a significant antiradical scavenging activity in comparison with the ascorbic acid used as control, the IC_50_ value noted was 20 mg/mL [34]. At the same level, a study on antioxidant activity using antiradical scavenging activity and the ferric reducing power of the methanolic extract showed that this organic extract is endowed with a weak antioxidant activity when tested in comparison with the ascorbic acid used as a positive control [35] (Table 1). The *NS* oil (NSO) was indicated to have an important antioxidant activity, the IC_50_ value for the DPPH• was 3.8 mg/mL, the ABTS• + 4.7 mg/mL, and for the ferric reducing power assay 0.8 mg/mL. While no effect was observed in the β-carotene bleaching test [36]. Besides the seed’s chemical composition, the methanolic extract of the aerial part is also rich with several chemical compounds of flavonoids nature. The evaluation of their antioxidant activities was evaluated using two methods DPPH and 2,2’-azino-bis(3-ethylbenzothiazoline-6-sulfonic acid (ABTS) assays. The results obtained show that among the 12 isolated molecules only five molecules (magnoflorine, nigelflavonoside, quercetin sphorotrioside, kaempferol-3, 7-diglucoside, and rutin) were active with an IC_50_ ranging from 95.18 µM to 197.8 µM for DPPH, while for ABTS the IC_50_ varies from 95.18 µM to 247 µM [20] (Table 1). A comparative study showed that the water-soluble extract (IC_50_ = 33.32 mg/mL) gave a very important antiradical activity in comparison with the oil extract (IC_50_ = 73.66 mg/mL), while the ascorbic acid gave the lowest IC_50_ (4.28 mg/mL) [37]. The evaluation of the free radical scavenging activity and ferric reducing power of native and roasted *NS* seeds showed a significant elevation of the antioxidant potentials whether native, pan or microwave roasting [38] (Table 1). 

#### 4.1.2. In Vivo

**Figure 6:**
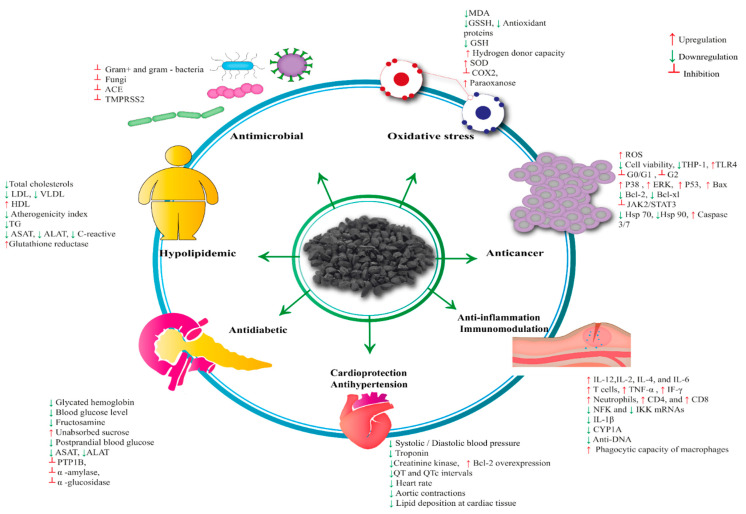
Comprehensive summary of the *NS* pharmacological activities.

The supplementation of rats with *NS* seeds was able to cause an increase in catalase (CAT) activity and the total antioxidant activity in comparison with the control [29] (Figure 6). In a clinical study, the antioxidant activity tested in vivo on healthy subjects by a daily administration of the aqueous extract (200–250 mL) during five days indicated a non-significant decrease on the sixth day of the MDA, and a non-significant increase of superoxide dismutase (SOD), while a significant increase of the erythrocyte glutathione GSH was noticed [31] (Table 1). A supplementation of rats with NSO or with TQ has induced an increase of the ceruloplasmin which represents an extracellular antioxidant responsible for the reduction of the Fe^2+^ to Fe^3+^. The administration of both the NSO and TQ were found to have a preventive effect of renal tissues against the radiations and this by an elevation of the paraoxonase on one side and a decline of the hydroperoxide lipid on the other side. The *NS* seeds induced a reduction of the total antioxidant status, and the oxidative stress index at the renal level of irradiated rats. All these results confirm those obtained in the in vitro studies [41] (Table 1). The streptozotocin (STZ)-induced diabetes model was used to assess DNA damage by comet assay, which measures STZ-induced breaks directly, and also to evaluate the protective effect of *NS* against the STZ damage. A daily administration of 500 mg/kg of *NS* ethanolic extract produced a significant decrease of the induced damage at the level of the DNA, a decline at the level of the lipid peroxidation, as well as an observed elevation of the SOD activity [40]. Similarly, the treatment of rats with black cumin oils proved their antioxidant activities which are characterized by a decrease of the MDA and oxidized glutathione (GSSG), as well as an increase of the hydrogen donor capacity [39] (Table 1).

### 4.2. Antimicrobial Activity/Antibacterial Activity

#### 4.2.1. In Vitro

The obtained fractions from the methanolic extract of *NS* showed an important antibacterial activity on different strains such as *Staphylococcus aureus*, *E. coli*, and *Pseudomonas aeruginosa* (Figure 6). Also, this inhibition potential was confirmed on multi-drug resistant bacteria like the *Staphylococcus saprophyticus* and *Staphylococcus epidermis* [35]. A synergic effect was observed when testing the antibiotics (ATB) with the *NS* oils obtained with the cold press technique on the methicillin-resistant S. aureus (MRSA) which showed potentiation of the effect exerted by the ATB. This combination of the oil with ATB especially with ‘Augmentin’ showed a better bactericidal effect on the MRSA. The scanning electron microscope revealed a membrane deformation of the bacterial cell [42]. Concerning the aqueous extract obtained by a decoction of the *NS*, seeds indicated an antibacterial potential at a concentration of 100µg/mL against gram-positive (*Micrococcus luteus, S. aureus, Bacillus subtilis*), and gram-negative bacteria (*Agrobacterium tumefaciens, Salmonella setubal, Enterobacter aerogenes*) [43] (Table 2). 

The n-hexane extract showed promising antibacterial activity when tested on isolated clinical *S. aureus*, also on MTCC bacteria *S. aureus*, and *Salmonella typhi* [45]. A study assessed on TQ showed that the molecule has a great capacity to inhibit the bacterial growth for several bacterial strains. The treatment by different concentrations of the same molecule showed significant inhibition of the biofilm formation. It has also been demonstrated that the molecule showed a highly synergic effect in the presence of the antibiotics and this against gram-positive and gram-negative bacteria [46]. Similarly, the effect of TQ, methanolic extract, and NSO (extracted and commercial) showed better inhibition of the *Bacillus subtilis*, and *Bacillus licheniformis* [47]. 

The n-butanol extract obtained from the seeds of *NS* exhibited a strong antibacterial activity on *P. aeruginosa*, *Klebsiella pneumoniae*, and *Acinetobacter baumannii,* with a minimal inhibitory concentration that varies between 0.25 and 1 µL/mL. On the contrary, the same extract was inactive on *E. coli* and *S. aureus*. The antibacterial effect of butanol extract against the bacterial strains responsible for food poisoning and nosocomial infection could be due to its richness with fatty acids and terpenoid compounds [51]. The nanoparticles formed by green synthesis from the silver nitrate, plus the *NS* aqueous extract gave an antibacterial activity with different inhibition diameters 13 mm for *S. aureus* and 6 mm for the *E. coli* at a concentration of 20 mg/mL [50]. On the other hand, the tested NS EO was found to be inactive on multidrug-resistant bacteria isolated from poultry like the *Salmonella gallinarium* and *Salmonella enteriditis* [52]. In the same way, the black cumin EO and the different compounds such as thymol, TQ, carvacrol, and β-cymene were found to be very effective on *Chlamydia trachomatis* one of the principal causes of sexually transmitted diseases [53]. Finally, in a comparative study assessed by Dalli et al., 2021 [8] the *NS* EOs originating from four countries have shown their antibacterial effect on MDR-bacteria such as MRSA, extended spectrum beta-lactamase, *A. baumannii*, and *P. aeruginosa* which was attributed to the different bioactive compounds present in the different volatile compounds obtained by hydrodistillation. Another study on the in vitro inhibitory capacity of the *NS* EO, thymol, and TQ showed an inhibitory capacity against *Fusobacterium nucleatum* associated with *Actinomyces naeslundii* [48] (Table 2).

#### 4.2.2. In Vivo

The NSO was found to be very effective on bacterial infection after an intraperitoneal injection of *Aeromonas hydrophila* and *Pseudomonas fluorescens* to *Oreochromis niloticus*. This administration of NSO was accompanied by a decrease in the cytochrome P450 1 A (CYP1A) expression which increases in the case of infection as a response to oxidative stress and for detoxification of the organism [49]. In a clinical study, the supplementation of the *NS* with the quadritherapy demonstrated a beneficial effect on the eradication of *Helicobacter pylori*, also an amelioration of different parameters such as body weight, and the body mass index [44]. Moreover, an addition of essential oils to stocked boilers meat gave satisfactory results, leading to a reduction in the total number of bacteria and cold-resistant bacteria while also helping to maintain the nutritional value of the meat [54]. These results were in accordance with those obtained after the pre-treatment of the meat with the commercial essential oil which induced a significant decrease of *Bacillus spp.* This latter was found to be very sensitive to the EO and the *NS* powder [55] (Table 2). 

### 4.3. Antiviral Activity

The study of the effect of black cumin oil on patients who tested positive for COVID-19 with mild symptoms shows that this oil at a dose of 500 mg/kg was able to improve the average number of days to cure in the NSO group, around 10.7 days compared with the control group’s 12.4 days. The percentage of patients cured in the NSO group was significantly higher than in the control group, with 57 patients (63%) versus 32 patients (35%), respectively [60]. 

Several studies have been realized computationally (in silico) for a better understanding of the antiviral activity assessed on SARS-CoV 2. A study performed in silico showed that TQ may exhibit inhibitory activity against the SARS-CoV 2 protease [61], and has been mentioned as being able to induce oxidation of the virus, protecting cells by modulating endosomal pH [62]. The molecular docking study confirms the same results obtained by Koshak et al. 2021 [60] which suggest that TQ can act against COVID-19 by inhibiting the angiotensin-converting enzyme 2 (ACE2) which inhibits the interaction of viral proteins. Several hypotheses suggest that nigellimine, an alkaloid present in NS seeds, can block the entry of SARS-CoV 2 through an inhibitory activity on ACE2 and it has also been reported that co-administration of hydroxychloroquine (HCQ) with black cumin seeds reduced the toxicity and potentiated the antiviral action of HCQ against COVID-19 [63]. In the in-silico study by molecular docking of nine bioactive compounds from black cumin, the theoretical results show that four of them gave good binding affinity towards RNA-dependent RNA polymerase. For example, α-hederin gave a binding affinity that is higher than that demonstrated by Remdesivir used as an antiviral agent. While TQ, nigellicine, and nigellidin gave an affinity low to that recorded by α-hederin but was close to that of Remdesivir [64]. The inhibitory power of several bioactive compounds was tested in silico on transmembrane serine protease 2 (TMPRSS2) which is an enzyme present at the level of pneumocytes II and in the case of the presence of the virus this enzyme truncates (amputates) the spike protein which thus facilitates its endocytosis. The results obtained show that carvacrol was able to theoretically inhibit TMPRSS2 more than Comstat used as a control, while thymol, given in silico, always produced a low activity [65]. A study conducted by Sajjad Ahmad et al., showed that nigellone, also called dithymoquinone, was able to theoretically inhibit all four crucial targets of coronavirus [66], while nigellidine and kaempferol, which are two compounds present at the seed level, also showed a high affinity with COVID-19 C19MP proteases [67] (Table 2).

### 4.4. Antifungal Activity

The fractions obtained from methanolic extract were found to exhibit an antifungal activity when tested on *Trichophyton* sp., *Candida albicans*, *Candida tropicalis*, *Candida krusei*, *Penicillium* sp. and *Aspergillus niger* [35] (Figure 6). An MIC value of 25 and 12.5 mg/mL respectively of *Candida albicans* and *Candida parapsilosis* was noted when testing the NS ethanolic extract [57]. The aqueous extract tested on fungal strains has been mentioned to be effective when evaluated on different Mucor fungal species such as *Fusarium solani*, *Aspergillus fumigatus*, *Aspergillus flavus*, and *Aspergillus niger,* with the inhibition percentages ranging from 30 to 70% [43]. Furthermore, an isolated peptide from the NS seeds named nigellothionines has been found to exhibit an antifungal activity toward *A. flavus*, *A.fumigatus*, and on *A.oryzae* [56] (Table 2). Meanwhile, n-butanol extract has been found to have an antifungal action against *Candida albicans*, *Candida krusei*, and on *Candida parapsilosis* with an MIC of 0.125 and 0.5 µL/mL. The terpenoid compounds and the fatty acids could be the responsible elements for the antifungal effect [51]. 

The different fractions obtained after fractionation by different organic solvents from the methanolic extract of the vegetative parts of nigella have been tested for their antifungal power and this on two strains *Fusarium oxysporum,* and *Macrophomina phaseolina.* The obtained results indicate that the ethyl acetate fraction was able to inhibit the growth of the two used fungi at a concentration of 50 mg/mL; furthermore, the chloroform fraction has the same ability to inhibit *M. phaseolina* at the same concentration of 50 mg/mL. Meanwhile, the n-hexane and the n-butanol extracts induced a significant inhibition that reached the level of 88% at 50 mg/mL, finally, the aqueous fraction has the lowest inhibitory activity of about 5% [59] (Table 2).

#### In Vivo

An in vivo study on the therapeutic potential of black cumin was verified on female rats inoculated with *Candida albicans* which is considered as the major cause of vulvovaginal candidiasis. The results obtained reveal a significant decrease in the number of fungal colonies after treatment with *NS* extract at a dose of 6.6 mL/kg [58] (Table 2).

### 4.5. Antiparasitic

Concerning the antiparasitic activity of the plant, it has already been mentioned by El-Sayed et al. in 2019 [68] that TQ has a great ability to inhibit, in vitro, different parasites such as *Babesia bovis*, *Babesia bigemina*, *Babesia divergens*, *Theileria equi*, and *Babesia caballi*. The combination of TQ and the diminazen aceturate on *Babesia bovine, Babesia equine,* and *Theileria* parasites gave a considerably high effect. The per os administration and intraperitoneal injection of TQ at a dose of 50 and 70 mg/kg to mice induced the growth inhibition of *Babesia microti* [68]. 

### 4.6. Anticancer Activity

More than the antioxidant activity mentioned before, the formulation between the *NS* extract and honey gave an inhibition of the growth of ovarian cancer cells PA-1, and this in a dose-dependent manner [34]. In the same context, the aqueous extract has demonstrated a cytotoxic effect on brine shrimp *Artemia salina*, the obtained results indicate the presence of a cytotoxic effect with an IC_50_ value equal to 284.9 mg/mL [43]. The nigellothionine tested on B16 cells (mice melanoma), HTC-116 (human adenocarcinoma), and human postnatal fibroblasts (HPF) showed a decrease in the cell viability of the three used cell lines [56] (Table 3). 

To evaluate the potential of *NS* seeds as an application to the oral mucosa in the prevention of oral cancer. An aqueous extract of whole seeds of *NS* was prepared, mimicking the case of chewing the seeds within the mouth. The results obtained indicated that 5% of the diluted extract induced inhibition of cancer cell growth. The chemical analysis of this extract showed the presence of a small amount of TQ, while the α-hederin was the major compound. The α-hederin alone at a dose of 20 µg/mL induced an effect very similar to that of the aqueous extract on oral cancer cells, which suggests that this molecule is the element responsible for the anticancer activity [69]. Another study that focuses on testing TQ on liver cancer cells SK-Hep 1 demonstrated that this molecule can activate cell death by the activation and elevation of P38 phosphorylation and by an increase of extracellular signal-regulated kinases (ERK) (Figure 6). The P38 inhibition was accompanied by the cessation of TQ activity [79]. Similarly, TQ inhibited breast cancer cells MDA-MB 231, and inhibited their proliferation at low doses of 50 and 100 µM (Table 3). 

Ferulic acid, a phenolic compound present in the seeds of *NS* gave, an antiproliferative action at a concentration of 450 µM. On the other hand, no effect of the ferulic acid was noted at a concentration of 250 µM, but its combination with the 25 µM of TQ induced a significant decrease in cell proliferation [73]. Concerning the virgin oil, rich with volatile compounds of *NS*, obtained by cold press and tested on MCF5 and A324, this showed an apoptotic activity that was dose-dependent with an LC_50_ of 1.6 µg/mL and 1.3 µg/mL for MCF5 and A325, respectively. Whereas, the black cumin oil without volatile compounds gave no cytotoxic effect even at a high dose of 3.09 µg/mL, and this despite the presence of TQ which suggests the intervention of other molecules in the obtained pharmacological property [76] (Table 3). 

The plant melanin was obtained from *NS* seeds tested on THP-1 cells (a human monocytic cell line derived from acute monocytic leukemia) and also on HEK293 embryonic kidney cells. The obtained results indicate that the exposition of THP-1 to 500 µg/mL of plant melanin caused a significant decrease in the cell viability to 90% as well as a cell arrest at the level of G0/G1. This registered effect was accompanied by an augmentation of TLR4 which is known as the site of apoptosis induction. While no effect was observed on HEK293 at lower doses, at a dose of 500 µg/mL a cell viability reduction of about 80% was noticed; in addition to a blockage of the cell cycle at the G2 phase. No TLR4 expression was observed at the level of HEK293 cells [70]. The cytotoxic effect of the *NS* melanin was confirmed in a study performed by Al-Obeed et al. in 2020 [71], which demonstrated that the plant melanin was endowed with an inhibitory action of the colorectal adenocarcinoma HT29, and on the colorectal cancer metastasis SW620. In addition to this cytotoxic effect there was an elevation of the reactive oxygen species, modulation of the MAPK pathway by activation of the JNK pathway, and an inhibition of the ERK phosphorylation. The supplementation of Balb/c mice by 500 mg/kg of “NSO” only or with “NSO + cisplatin” induced an increase in the polychromatic erythrocyte (PCE) which reflects a rapid recovery of bone marrow performance. This indicates the cytoprotective and reno-protective effects of black cumin seed oils [72] (Table 3).

TQ was found to induce apoptosis of renal human cancer cells CaKi-1 at a concentration of 25 µM, an effect that was accompanied by an elevation of pro-apoptotic markers such as P53, Bax, and Cytochrome C. On the counterpart, a reduction of the expression of anti-apoptotic elements Bcl-2 and Bcl-xl was noticed. In the same study, it is mentioned that treatment with TQ-induced inhibition of JAK2/STAT3 pathway phosphorylation induces inhibition of different elements such as cycline D1, cycline D2, and survivine (Figure 6) which are mainly involved in cell proliferation and the fight against apoptosis. TQ effectively protected the localization of phosphorylated STAT3 in the nucleus and thus inhibited its binding to DNA. In addition to its inhibitory role, TQ also caused an increase in reactive oxygen species (ROS) inside CaKi-1 cells. The antitumor effect was also verified by intraperitoneal injection of CaKi-1 tumor cells and TQ into BALB/c mice. The results obtained show a reduction in the number of injected cancer cells compared with the control [74] (Table 3).

The treatment of lung cancer cells NCI-H292 with an herbal combination formed by NS seeds, *Hemidesmus indicus*, and *Smilax glabra* demonstrated that the ethyl acetate extract of the formulation showed a better cytotoxic effect. The induced effect was accompanied by changes at the level of the cell morphology such as a reduction of the cell volume and the formation of bulbs. Also, a tiny fragmentation at the DNA level was observed after treatment with the ethyl acetate extract at a concentration of 300 µg/mL. At the molecular level, it has been noted that an augmentation of capase 3/7 activity at 50 µg/mL with a subexpression of Bac and P53 induced apoptosis of cancer cells. On the counterpart, a downregulation was registered of heat shock proteins (Hsp) 70 and 90 [77] (Table 3). 

The study by Kordestani et al. in 2020 showed that the *NS* hydroalcoholic extract was able to inhibit the cancer cell proliferation of MCF-5 in a dose-dependent manner, with the registered IC_50_ after 24 h of treatment being 3.29 mg/mL [78]. The sapindoside B extracted from black cumin seeds presented a cytotoxic action against HCT116, AGS, and on HCC-LM3 with an IC_50_ that was lower than 10 µM, but was moderately active on A549, H1299, H460, HGC27, and HepG2 with an IC_50_ ranging from 11.93 to 20.05 µM. The extracted saponin was inactive on gastric cancer cells MGC830 [75] (Table 3).

### 4.7. Anti-Inflammatory and Immunomodulatory Activity

Concerning the anti-inflammatory activity, NSO injection at different doses to rats with carrageenan-induced edema in the hind paw causes significant suppression of the edema, this effect has been attributed to the inhibition of eicosanoid and lipid generation [80]. Gholamnezhad et al. investigated the immunomodulatory effect of NS on Wistar rats after injection of 10% PHA (phytohemaglutinin), where an improvement of the animal weight was observed. The supplementation of the animals with a dose of 50 g/kg caused an increase in the size of the spleen which is an organ responsible for the clearance of particles that hurt the body. The antioxidant effect of the plant can be attributed to its immunostimulant activity which is manifested by the increase of IL-12 which in turn stimulates the production of TNF-α, IF-γ, and also CD8 in the spleen of treated rats [81] (Table 4). 

Acute treatment with black cumin oil by a dose of 2 and 4 mL/kg showed anti-inflammatory effects in comparison with diclofenac used as control, while no anti-inflammatory activity was observed in sub-acute treatment with black cumin oils [39]. TQ was signaled to protect against subgingival inflammation, a chronic periodontitis pathology, and achieved this by inhibiting the production of TNF-α [48]. Kordestani et al. have mentioned the ability of the hydroalcoholic extract to decrease the expression of NFK and IKK mRNAs (Figure 6) which explains that the anti-inflammatory effect of the plant is associated with a reduction in the expression of these genes [78]. Black cumin oils were also able to decrease the expression of IL-1β and also CYP1A [49] (Table 4).

To test the effect of *NS* polysaccharides (NSSP), mice were subjected to intraperitoneal injection of cyclophosphamide CTX to induce immunosuppression. Gavage of mice with different concentrations of polysaccharides induced protection of thymus and spleen against CTX-induced damage. It has been mentioned by the same authors that an increase of lactate dehydrogenase and acid phosphatase indicates the immunomodulatory effect exerted. The administration of CTX was accompanied by a decrease in total antioxidant capacity, SOD, as well as a decrease in the activity of endogenous antioxidants such as CAT, while an increase in MDA was also recorded. All these effects were mitigated by the administration of polysaccharides where an increase in total antioxidant capacity, SOD, was recorded. This administration induced an increase in CAT activity accompanied by a decrease in MDA. The results of this experiment show that the high dose group of NSSP significantly increased the levels of IL-2, IL-4, and IL-6 in the serum of mice (Figure 6), decreased the level of TNF-α, and regulated the level of cytokines to exert immunomodulatory effects. PTEN is a homologous negative regulator of the phosphatase-tensin gene, and its deregulation and deletion lead to the activation of the PI3K signaling pathway. It has been found that the administered group exerted immunomodulation by up-regulating PI3K protein expression and down-regulating PTEN expression, increasing antioxidant enzyme activity and decreasing ROS and TNF-α, a pro-inflammatory factor. Decreasing the expression of PTEN, a negative regulator of PI3K, by activating the PI3K/Akt signaling pathway to regulate FoxO1 factor, inhibited TLR4/NF-B and the expression of pro-inflammatory cytokines, increasing the release of anti-inflammatory factors and thus exerting the immunomodulatory effect [85] (Table 4). 

Systemic lupus erythematosus (SLE), is a systemic disease characterized by the production of anti-nuclear antibodies directed in particular against native DNA. The effect of *NS* ethanolic extract was tested on lupus mice by pristane. The results obtained show that anti-DNA tends to decrease with increasing dosage of ethanolic extract. These results suggest that black cumin was able to improve the Th1 and Th2 balance, which is accompanied by a reduction in immune hyperreactivity. In the case of (SLE) a decrease of Th1 and an increase of Th2 results in excessive activation of β cells and therefore an increase of autoantibodies including anti-DNA. Treatment with a dose of 4.8 g/kg/d for one month increased regulatory T cells in lupus mice. Thus, the ability of black cumin to increase regulatory T cells and decrease Th17 induces a return to a steady state which explains the potential of black cumin seeds to repair immune function in SLE (Guritno et al., 2021). The same results are mentioned by Gholamnezhad et al. after administration of 200 mg/kg of black cumin which may balance the Th1/Th2 cytokine profile of the spleen in favor of Th1, especially in unstimulated overtraining conditions [81] (Table 4). 

In addition to its effect on improving the antioxidant activity in patients with β-thalassemia, supplementation with *NS* seeds (2 g/d) for 3 months boosts cell-mediated immunity which is characterized by an increase in the number of neutrophils and also an increase in CD4 from 1319.8 to 2007.6 cells/µL. While CD8 increased from 727.09 before administration to 1145.3 cells/µL [82]. Finally, it has been noted that hydroalcoholic extract induced an increase of the phagocytic and lethal capacity of macrophages towards *S. aureus* in comparison with control [83] (Table 4).

### 4.8. Cardioprotective and Antihypertensive Activity

The administration of NSO to patients suffering from hypertension over eight weeks induced a reduction in systolic and diastolic blood pressure [86] (Figure 6). The pre-treatment of rats over 14 days by a dose of 4 mL/kg/day produced a reduction in the increase of troponin and also that of creatinine kinase after their increases by an injection of isoproterenol (ISO). Pre-treatment with this oil also caused a significant reduction of aspartate aminotransferase (ASAT) after acute induced myocardial infarction. Also, the NSO has shown no influence on the complex QRS or the PR interval. A reduction in the prolongation of QT and QTc intervals was observed and the prevention of a reduction in R-wave amplitude induced by ISO [30] (Table 5). 

The supplementation of healthy obese and overweight subjects with 2 g/day demonstrated a decrease in systolic pressure while no effect was observed on diastolic pressure [89]. Similarly, a study of cardiac hypertrophy showed an increase in cardiomyocyte diameter in sedentary rats, under-exercised rats, and also in rats under “exercise + *NS*”. This increase was accompanied by a significant elevation of growth hormone GH and Akt. The action of GH is mediated by the release of IGF-I from various tissues such as the liver, myocardium, and skeletal muscle. IGF-I, which is a structurally similar peptide to insulin, is released into the myocardium in a paracrine way, then binds to transmembrane tyrosine receptors in cardiomyocytes, and causes activation of the PI3K-Akt pathway to mediate cardiac hypertrophy. In this study, IGF-I was elevated only in the exercise group while no difference was observed in the “*NS* + exercise” group. This suggests that the administration of *NS* may cause the intervention of another IGF-I analog. Thus, Ns-induced cardiac hypertrophy is mediated by the GH-IGF I-PI3P-Akt pathway [94] (Table 5). 

In another study, Azithromycin was able to induce myocardial necrosis, fibrosis, and apoptosis as well as increased creatinine phosphokinase (CPK), lactate dehydrogenase (LDH), TNFα, and malondialdehyde and smooth muscle α-actin. Cotreatment with NSO significantly reduced CPK, LDH, MDA, and TNFα levels, preserved cardiac morphology, and decreased Caspase-3 and α-SMA immunoreactivity compared with the Azithromycin group [87]. Pei et al. demonstrated the cardioprotective effect of TQ which was shown to be responsible for the decrease of lipid deposition at the cardiac tissue [92]. The hydroalcoholic extract was responsible for the significant reduction of aortic contractions subjected to high concentrations of phenylephrine, while it was also observed that this extract gave the best aortic relaxation effect. Thus, the administration of hydroalcoholic extract improves the reactivity of the aorta to vasoconstrictor and vasodilator agents [95]. Likewise, black cumin seeds were able to decrease systolic and diastolic blood pressure in hypertensive rats, as well as their combination with amlodipine, showed better control of blood pressure and also a reduction of heart rate [90] (Table 5). 

The cardioprotective effect of black cumin oils was tested on female rats with streptozotocin-induced diabetes. The results mention that the NSO group showed a normal histological structure while in the untreated diabetic group there was observed a mild myositis, hyaline degeneration, and extensive Zenker necrosis. Concerning the diabetic group treated with NSO, hyperemia and hyaline degeneration in some muscle cells was observed, an effect that was accompanied by an elevation of Bcl-2 overexpression thus improving cell survival by suppressing apoptosis. Thus, NSO was able to exert a cardioprotective effect in diabetic rats [88]. In the same context, the long-term administration of TQ (two months) induced an inotropic effect characterized by maximal tension that is mediated by increased sensitivity of contractile proteins to Ca^2+^. No effect was observed of TQ on half-relaxation time and myocardial output [93] (Table 5). 

The administration of black cumin seeds at a dose of 300 mg/kg/day to hypertensive rats (L-NAME) showed a decrease in systolic blood pressure (SBP) by 8.7% and diastolic blood pressure (DBP) by 10.52%. While the maximum decrease value was 16.7%, recorded after 4 h of administration. The combination of L-NAME, Nigella seeds, and metoprolol induced a decrease in SBP by 16.1% and in DBP by 23.26%. The maximum value observed after 4 h of administration was 25.6% for SAP and 31.72% for DBP. There was a decrease in mean arterial pressure (MAP) of 9.71% after treatment with “*NS* + L-NAME”, while treatment with “L-NAME + *NS* + Metropolol” induced a decrease in MAP by 11.43% [96] (Table 5).

### 4.9. Antidiabetic Activity

It has been shown that different organic extracts of black cumin such as hydroacetone extract have the ability to inhibit α-amylase [97] (Figure 6). Also, the consumption of 5 g/day of black cumin for six months caused a decrease in glycated hemoglobin [98]. On the other hand, the nanoparticles formed from silver nitrates showed inhibitory activity on both α-amylase enzymes and α-glucosidase which was higher than that obtained by aqueous extract or even by acarbose used as a control [99]. The hydroalcoholic extract (50%) was tested on rats with streptozotocin-induced diabetes. The results obtained show that this extract can decrease fasting blood glucose [95]. As reported by Dalli et al. [19], the different fractions obtained by the soxhlet apparatus (Aqueous, MeOH, EtOH, Hex) induced important inhibition not only for the α-amylase (in vitro) but also for the intestinal glucose absorption (in situ) (Table 6). The combination of TQ and metformin (50/200 mg/kg) caused a decrease in blood glucose level by 41.3%. The administration of the combination of TQ (50 or 100 mg) with metformin (1000 mg) caused some adverse effects in some volunteers, among the effects mentioned, were diarrhea and abdominal pain. For those who completed the study a decrease in glycated hemoglobin was observed after three months. A greater reduction in fasting blood glucose and postprandial blood glucose was also observed in both groups compared with the control group [100]. The study of the inhibitory activity of a hydroalcoholic combination (70%) of *Morinda citrifolia*, *Trigonella foenum-graecum*, and *NS* on pancreatic α-amylase showed that this combination at different doses was able to inhibit the activity of the enzyme. Thus, it was mentioned that the dose of 140/70/140 mg/kg was the most effective among the doses studied and it was shown that this dose was able to inhibit a-amylase at low doses with an IC_50_ 35.7 µg/mL in comparison with acarbose used as control (83.07 µg/mL). The study of this combination in vivo confirmed the results obtained in vitro and it was mentioned that all three doses were able to decrease the blood glucose level, especially dose 2 which gave the best anti-diabetic effect followed by dose 1 [101] (Table 6). The n-hexane extract obtained by maceration from black cumin seeds was able to inhibit the activity of two key enzymes in type 2 diabetes α-amylase with an IC_50_ 403.5 µg/mL and α-glycosidase with an IC_50_ of 74.7 µg/mL. These obtained results were attributed to the different chemical compounds present such as linoleic acid and palmitic acid [36]. A study carried out on overweight, type 2 diabetes, and overweight + type 2 diabetes volunteers by a combination of black cumin seeds and fenugreek induced a decrease in glycated hemoglobin which correlated positively with a decrease in liver enzymes [102]. From the methanolic extract of the aerial part of *NS*, several chemical compounds were isolated and tested on intestinal α-glucosidase and phosphatase tyrosine kinase (PTP1B), which is considered a negative regulator in insulin signal transduction. Hederagenin, flaccidoside III, quercetin-3-gentiobiosides, magnoflorin, nigelflavonoside B, nigelloside, quercetin sphorotrioside, kaempferol-3, 7-diglucoside, Kaempferol 3-O-rutinoside, and rutin were able to inhibit α-glucosidase at different concentrations with an IC_50_ ranging from 256.7 ± 3.7 µM to 331.9 ± 1.6 µM. The compound named 3-O-[α-L-Rhamnopyranosyl-(1-2)-α-l-arabinopyranpsyl] hederagenin was the only compound able to inhibit phosphatase tyrosine kinase with an IC_50_ of 91.3 ± 2.5 µM [20] (Table 6). The ethanolic extract (300 mg/kg) of black cumin seeds, when tested on rats with streptozotocin-induced diabetes, revealed a non-significant decrease in blood glucose, while the tested extract induced a significant decrease in fructosamine, as well as an improvement of the lipid profile. Histologically, only a few samples showed pycnotic changes (early necrosis) in the pancreas. On the contrary, the rest of the tested samples showed an improvement in the number and morphology of islet cells, which were quite normal with a slight interstitial dilation of the blood vessels, and a slight perivascular dilation [103]. Regarding the glucose tolerance test, the methanolic extract at a dose of 500 mg/kg was able to decrease blood glucose. A suppression of sucrose-induced hyperglycemia after treatment with the same extract, as well as an increase in unabsorbed sucrose, was observed in the stomach, upper and mid intestine after 30 min of administration, after 1 h in the lower intestine, and in the cecum and large intestine after 2 h. The methanolic extract was not only able to inhibit glucose absorption but also to inhibit the enzyme disaccharidase [104]. The study carried out on volunteers revealed that there is no effect after supplementation with black cumin seeds on insulin secretion [105]. Aqueous and ethanolic extracts exerted an anti-glycation effect at different doses [106], these same results were observed after supplementation of rats with hexane extract, where a decrease in glycated hemoglobin percentage was reported [107]. Treatment with a combination of three medicinal plants *Artemisia sieberi*, *NS,* and *Teucrium polium* uncovered a significant decrease in blood glucose in treated rats. The mentioned effect was accompanied by a decrease in intrinsic antioxidant enzymes such as SOD and CAT [108]. Treatment of rats with a combination of hydroalcoholic extracts (water 80%–MeOH 20%) of *Trigonella foenum-graecum*, *NS*, *Zingiber officinale*, *Olea europea*, *Fraxinus ssp* for two months protected the functioning of the pancreas. Ultrastructure study by transmission electron microscope (TEM) showed that the acinar cells of the group treated with the herbal combination had normal ultrastructure, with normal cell activity and nuclei, and a well-developed rough endoplasmic reticulum with abundant zymogen (Proenzyme) granules. This treatment with a mixture of plants allowed the protection of the pancreas against the different degenerative effects caused by diabetes induced by alloxan injection. While, the untreated diabetic rats presented disturbances in the arrangement of pancreatic acinar cells, a decrease in secretory granules, vacuolation of the cytoplasm, and pycnotic nuclei were observed (Table 6). This confirms the pancreato-protective effect of this combination [109]. These results were in agreement with those obtained by Aboul-Mahasen & A. Alshali who showed that supplementation with black cumin seeds unveiled regeneration of exocrine and endocrine parts of pancreatic tissues of hyperlipidemic rats fed with *NS* seeds [110].

### 4.10. Anti-Obesity and Dyslipidemic Activity

Concerning the effect on the lipid profile, treatment with the NSO induced a decrease in total cholesterols, low-density lipoproteins (LDL), and malondialdehyde (MDA). While this administration was accompanied by an increase in high-density lipoproteins (HDL), and glutathione reductase [30], the results obtained were in agreement with those obtained by Bonab et al. [107]. The supplementation of obese and overweight healthy women with 2 g/day for eight weeks induced an increase in HDL. On the other hand, there was a decrease of atherogenicity index and LDL and also of serum glutamic-oxaloacetic transaminase [89] (Table 7). 

The supplementation of overweight, type 2 diabetes, and overweight + type 2 diabetes volunteers with a combination of black cumin seeds and fenugreek induced a decrease in LDL, TG, and VLDL and also atherogenicity index [102]. It was also mentioned that TQ was responsible for the decrease of total cholesterol, LDL, and also C-reactive proteins [92] (Table 7). 

The treatment of rats for six weeks with the hydroalcoholic extract of *NS* induced a reduction of cholesterol, TG, and LDL, while an increase in HDL was reported. There was an improvement in the vasodilatory response to acetylcholine in the aorta of diabetic rats. The hydroalcoholic extract of *NS* prevented an increase of VCAM-1 in diabetic rats which in case of elevation could recruit monocytes to the sites of atherosclerotic lesions and play a crucial role in the initiation and development of inflammation. Thus, the decrease in VCAM-1 could reduce vascular inflammation and improve endothelium [111]. An investigation on the supplementation of volunteer patients with type 2 diabetes with a combination of *NS* and fenugreek (2 g:10 g/d) for two months showed a significant decrease in total cholesterol, LDL, MDA, and triglycerides compared with the control group. On the other hand, an increase in HDL was observed which was not significant compared to the baseline. The effect exerted by the combination of *NS* and fenugreek can be explained by a stimulation of carbohydrate metabolism, including the absorption of glucose by the cell, improvement of gluconeogenesis, increase in the rate of absorption from the gastrointestinal tract, and also an increase in insulin secretion [112] (Table 7). 

In addition to the demonstrated hypoglycemic effect, the combination of *Artemisia sieberi*, *NS,* and *Teucrium polium* had a hypolipidemic effect especially on cholesterol, LDL, and triglycerides as well as a decrease in the liver enzymes ALAT and ASAT. While an improvement was observed through an increase in HDL [108]. There was an improvement in biochemical parameters after feeding with black cumin seeds with a decrease in HDL, something which is in contradiction with later studies [110] (Table 7).

### 4.11. Toxicology

Several studies regarding NS demonstrate the non-toxicity of the plant which confer to it a large interval of safety. The per os administration of NSO (2 mg/kg) for 12 weeks showed a very low toxicity with an LD_50_ 28.8 mL/kg body weight. While the intraperitoneal administration gave an LD_50_ of 2.06 mL/kg body weight [113]. The acute toxicity of the different fractions obtained using the soxhlet apparatus showed no toxic effect even at higher doses (5 g/kg) [19]. Meanwhile, TQ administration to albino mice at a dose of 870 mg/kg body weight was highly toxic. The LD_50_ value was 794 mg/kg body weight when tested on rats [114]. A subacute treatment with TQ for a 90 days period demonstrated no physiological changes [115]. 

## 5. Conclusions and Future Perspectives

Since the 1950′s, NS phytochemicals and pharmacological properties have been studied exhaustively. In the present study we gathered almost of the published articles between 2019 to 2021. During this period, some illnesses were well described and their action mechanisms were well illustrated; on the other hand, other illnesses were less prioritized. In the last three years, scientists have started focusing more on the antiviral activity of the plant especially after the COVID-19 pandemic, also more studies were performed on NS nanoparticles obtained by green synthesis that are considered as promising agents in the treatment of various diseases. Furthermore, such a combination of the NS with other herbs was found to potentialize the pharmacological activity; while synergic effects with drugs used clinically have proved its impact, something which could significantly help in the development of effective drugs. Despite its poor availability, TQ was studied extensively, while since their chemical identification in 1995, no in vitro or in vivo studies were found in the literature on the pharmacological properties of the alkaloids of the NS seeds. In addition to that, almost all conducted investigations are limited to the pre-clinical level, further studies are required at the clinical level for a better translation of the obtained results on humans. Finally, this review could be considered as a compass for upcoming studies, and for future development of therapeutic agents in order to help reduce health complications. 

## Figures and Tables

**Table 1:** Summary of the antioxidant activities of the *NS.*

Extract/Compound	Methods	Test	Results	Reference
*** NS *** **seeds**				
*NS* supplementation (30 and 50 g/kg BW)	In vitro	DPPH (antiradical scavenging activity)	IC_50_ = 1.367 mg TE/g	[29]
	In vivo (Wistar rats)	Total antioxidant capacity (TAC)	- For 30 g/kg the TAC value was 1.0170 mmol/L - For 50 g/kg the TAC value was 1.31 mmol/L	
NSO (5 mg/mL)	In vitro	DPPH	IC_50_ = 12.713 mM T/100 g	[30]
		- DPPH - ABTS - FRAP	- IC_50_ = 3.8 mg/mL - IC_50_ = 4.7 mg/mL - IC_50_ = 0.8 mg/mL	[36,39]
	In vivo	Wistar rats	↓ MDA ↓ GSSH ↑ Hydrogen donor capacity	
MeOH extract (0.2/0.4/0.6 and 0.8 mg/mL)	In vitro	Erythrocytes exposed to H_2_O_2_	↓ MDA ↓ Antioxidant enzymes ↓ GSH Anti-hemolytic activity	[31]
EtOH extract (10, 30 and 50 µg/mL)	In vitro	Human umbilical vein endothelial cells H_2_O_2_	↑ GSH level ↓ Lipid peroxidation	[32,40]
	In vivo	Wistar rats	↓ DNA damages ↓ Lipid peroxidation ↑ SOD	
TQ (6 and 12 µM) (50 mg/kg/day)	In vitro	Irradiation HaCaT keratinocytes cells by the UVA	Inhibition of the cyclooxygenase 2 (COX2) via the activation of NrF2/ARE pathway.	[33,41]
TQ (6 and 12 µM) (50 mg/kg/day)	In vivo	Irradiation of Sprague Dawley rat kidney tissue	Arylesterase (not significant) ↑ Paraoxonase Sulfhydryl groups (not significant) ↑ Ceruloplasmin ↓ Lipid hydroxide	[33,41]
Aqueous extract + honey (25–125 mg/mL)	In vitro	DPPH	IC_50_ = 20 mg/mL	[34]
Aqueous extract (0.2/0.4/0.6 and 0.8 mg/mL)	In vivo	Human healthy subjects	↓ MDA (not significant) ↑ SOD (Not significant) ↑ GSH level	[31]
MeOH extract (100 µg/mL et 1000 µg/mL)	In vitro	- DPPH - FRAP (ferric reducing power)	- Inhibition percentage reached 40.37% at 1000 µg/mL - The percentage 48.4% at 100 µg/mL	[35]
Water-soluble extract	In vitro	DPPH	IC_50_ = 33.32 mg/mL	[37]
- Not roasted seeds - Pan-roasted seeds - Microwave roasted	In vitro	DPPH	Inhibition percentage of DPPH 87.76%, 86.14%, 87.11% respectively, for the different prepared seeds	[38]
		FRAP	The ferric reducing power percentage, 80.07%, 83.46%, 85.09% respectively for the different preparations	
**Aerial part**				
Magnoflorine (25, 50, 75 and 100 µM for DPPH test) (50, 100, 125 and 250 µM for ABTS test)	In vitro	- DPPH - ABTS	- IC_50_ = 71 µM - IC_50_ = 139.2 µM	[20]
Nigelflavonoside (25, 50, 75 and 100 µM for DPPH test) (50, 100, 125 and 250 µM for ABTS test)	In vitro	- DPPH - ABTS	- IC_50_ = 32.7 µM - IC_50_ = 95.18 µM	
Quercetin sphorotrioside (25, 50, 75 and 100 µM for DPPH test) (50, 100, 125 and 250 µM for ABTS test)	In vitro	- DPPH - ABTS	- IC_50_ = 35.5 µM - IC_50_ = 98.8 µM	
Kaempferol-3,7-diglucoside (25, 50, 75 and 100 µM for DPPH test) (50, 100, 125 and 250 µM for ABTS test)	In vitro	- DPPH - ABTS	- IC_50_ = 197.8 µM - IC_50_ = 247 µM	
Rutin (25, 50, 75 and 100 µM for DPPH test) (50, 100, 125 and 250 µM for ABTS test)	In vitro	- DPPH - ABTS	- IC_50_ = 39.6µM - IC_50_ = 129 µM	

↑: Upregulation; ↓: Downregulation.

**Table 2:** Summary of the antimicrobial activities of *NS.*

Extract/Fraction	Method	Bacterial Strains	Results	Reference
**Antibacterial Activity**				
*NS* supplementation + quadritherapy (2 g/day)	in vivo Clinical study on unhealthy volunteers	* Helicobacter pylori *	- Eradication of *H. pylori* - Amelioration of body weight, and the body mass index.	[44]
MeOH fraction	in vitro Agar diffusion method	- S. aureus - E. coli - P. aeruginosa - MRSA - S. epidermis (MDR)	All tested bacteria showed a susceptibility toward the methanolic fraction	[35]
*NS* + ATB (Augmentin^®^) (5 and 7.5 µg/mL for NS, 10 and 20 mg for Augmentin^®^)	in vitro Agar diffusion method	MRSA	Potentiation of the ATB activity, plus a membrane deformation	[42]
Aqueous extract	in vitro Agar diffusion method	- M. luteus - S. aureus - B. subtilis - A. tumefaciens - S. setubal - E. aerogenes	MIC at 100 µg/mL	[43]
Hexane extract (100 mg/mL)	in vitro Agar diffusion method	- S.aureus (clinical strain) - S.aureus (MTCC) - S.typhi (MTCC)	Inhibitory activity on the tested strains that was characterized by an inhibition diameter between 11.25 to 19 mm.	[45]
TQ (50 µg/mL) (1.25, 2.5 and 6 mg/µL) (1 µg/mL)	in vitro Microdilution technique, and agar diffusion method	- K.pneumoniae - S.epidermis (ATCC) - S.aureus - S.epidermis - Bacillus subtilis - Bacillus licheniformis - Chlamydia trachomatis	- Inhibitory activity of different gram+ and gram^–^ bacteria with a MIC ranging from 1.04 to 8.3 µg/mL. - Synergic activity in the presence of ATB - Inhibition of bacterial growth - Inhibition of bacterial growth - IC_50_ = 3.12 µM	[46,47,48]
		- Fusobacterium nucleatum associated to Actinomyces naeslundii	Inhibition of biofilm formation at a concentration of 0.1%	
NSO (1.25, 2.5 and 5 mg/µL) (7% mL/kg diet)	in vitro Agar diffusion method	- Bacillus subtilis - Bacillus licheniformis	A bacterial inhibition at a concentration of 5 µg/mL.	[47]
	in vivo	Injection of bacterial strains to *Oreochromis niloticus* - A.hydrophila - P.fluorescens	↓ CYP1A	[49]
Aqueous extract (20 mg/mL)	in vitro Agar diffusion method	- S.aureus - E. coli	- Inhibition diameter of about 13 mm. - Inhibition diameter 6 mm. At a concentration of 20 mg/mL	[50]
n-butanol extract (1.5, 2 and 2.5 µL/mL)	in vitro Microdilution method	- P. aeruginosa - K. pneumoniae - A.baumannii	A strong activity with an MIC value ranging from 0.25 to 1 µL/mL	[51]
		- E. coli - S.aureus	Inactive	
Essential oil (100 mg/mL) (0.1%) (0.25 and 0.5%)	in vitro Agar diffusion	- S. gallinarium - S. enteriditis	Inactive	[52,53]
	in vitro On infected Hela cells	* Chlamydia trachomatis *	IC_50_ = 0.009% *v*/*v*	
	in vitro On Stocked boilers meat	* Bacillus spp. *	↓ Total number of bacteria and also of the cold-resistant bacteria Total inhibition of bacterial growth	[54,55]
	in vitro Microdilution technique	- MRSA - Extended-spectrum *beta-lactamase* - *A.baumannii* - *P.aeruginosa*	An inhibitory potential with an MIC ranging from 3 to 20 µL/mL and an MBC value varies between (3 to 40 µL/mL).	[8]
Carvacrol (3.12, 6.25, 12.5, 25, 50 and 100 µM)	in vitro On infected hela cells	* Chlamydia trachomatis *	IC_50_ = 6.25 µM	[48,56]
Thymol (3.12, 6.25, 12.5, 25, 50 and 100 µM) (0.1%)	in vitro On infected hela cells	* Chlamydia trachomatis *	IC_50_ = 6.25 µM	
	in vitro	*Fusobacterium nucleatum* associated to *Actinomyces naeslundii*	Inhibition of biofilm formation at a concentration of 0.1%	
Cymene (3.12, 6.25, 12.5, 25, 50 and 100 µM)	in vitro On infected hela cells	* Chlamydia trachomatis *	IC_50_ = 3.12 µM	
**Antifungal activity**				
***NS* Seeds**				
MeOH extract	in vitro Agar diffusion method	- Trichophyton sp. - Candida albicans - Candida tropicalis - Candida krusei - Penicillium sp. - Aspergillus niger	- Inhibition of 66.67% of the tested fungi, while the 33.3% left were resistant. - *C. tropicalis* was the highly sensitive strain (18.0 mm), while *Penicillium* sp. Was less sensitive. - *Aspergillus niger* was not sensitive to the crude extract	[35]
EtOH extract	in vitro Microdilution method	- Candida albicans - - Candida parapsilosis	- MIC = 25 mg/mL - MIC = 12.5 mg/mL	[57]
n-butanol extract (1.25, 2 and 2.5 µL/mL)	in vitro Microdilution method	- Candida albicans - Candida krusei - Candida parapsilosi	MIC value between 0.125 and 0.5 µL/mL	[51]
Sodium carboxymethylcellulose *NS* extract (5 mg/mL at a dose 6.6 mL/kg)	in vivo	Injection of *Candida albicans* to female rats	Significant decrease of colonies at a dose of 6.6 mL/kg	[58]
Hexane, Ethyl acetate, MeOH, Chloroform extracts (Obtained by methanolic extract fractionation)	in vitro Agar diffusion method	- Trichophyton sp. - Candida albicans - Candida tropicalis - Candida krusei - Penicillium sp. - Aspergillus niger	- Hexane fraction was active on the different strains (2.0 to 50.0 mm) - Chloroform fraction was active on only 33%. - *Trichophyton* sp. and *Candida tropicalis* were considered as highly sensitive to the different fractions. - Ethyl acetate fraction had a zone inhibition ranging from (2.0 to 12.0 mm). - The different tested strains were found to be sensitive to MeOH fractions except for *A.niger*. - *Aspergillus niger* was not sensitive to the chloroform and ethyl acetate fractions.	[35]
Nigellothionines (concentrations ranging from 0.5 to 64 mg/mL)	*in vitro* Agar diffusion method	- A. flavus - A.fumigatus - A.oryzae	The zone inhibition ranged from 11 to 12.7 mm, and the MIC was 0.77 µM	[56]
**Aerial part**				
- Ethyl acetate fraction - n-hexane fraction - n-butanol fraction - Aqueous fraction (Obtained from methanolic extract fractionation) (concentrations ranging from 1.562 to 200 mg/mL)	Antifungal bioassay	- Fusarium oxysporum - Macrophomina phaseolina	The different fractions exhibited a biomass reduction at 50 mg/mL, except for the aqueous fraction that had a low activity	[59]

↑: Upregulation; ↓: Downregulation.

**Table 3:** Summary of the anticancer activities of *NS*.

Extract/Compound	Method	Cell Lines	Results	Reference
Aqueous extract (250, 500, 1000 mg/mL)	In vitro	Brine shrimp assay on artemia salina	Cytotoxic effect with an IC_50_ value equal to 284.9 mg/mL	[43]
*NS* + honey (concentrations ranging between 10 and 70 μg/mL)	In vitro	Ovarian cancer cells PA-1	Inhibition of the cell proliferation in a dose-dependent manner.	[34]
Aqueous extract prepared with mimicking the chewing process (1.25, 2.5 and 5%)	In vitro	Mouse squamous cell carcinoma cells SCC VII	5% of the diluted extract induced inhibition of cancer cell growth.	[69]
α-hederin (5, 10, 20, 40 and 80 μg/mL)	In vitro	Mouse squamous cell carcinoma cells SCC VII	The α-hederin alone at a dose of 20 µg/mL induced an antiproliferative effect.	
Nigellothionines (concentrations ranging from 0.1–50 μM)	In vitro	- B16 cells (Mice melanoma) - HTC-116 (Human adenocarcinoma) - Human postnatal fibroblasts (HPF)	- ↓ Cell viability of the three used cell lines. - The IC_50_ values registered were 0.30 for B16, 0.20 for HTC-116, and 0.55 for HPF.	[56]
*NS* melanin (concentrations ranging from 7.8–500 μg/mL) (concentrations ranging from 5–200 μg/mL)	In vitro	THP-1 cells a human monocytic cell line	- ↓ THP-1 cell viability to 90% as well as a cell arrest at the level of G0/G1 at 500 µg/mL. - ↑ TLR4	[70,71]
		- HEK293 embryonic kidney cells	- Reduction of 80% of HEK293 cells at500 µg/mL. - Blockage of the cell cycle at the G2 phase. - No TLR 4 increase.	
		- Colorectal adenocarcinoma HT29 - Colorectal cancer metastasis SW620	- ↑ ROS - Activation of the JNK pathway. - Inhibition of the ERK phosphorylation	
NSO (500 mg/kg/day)	In vivo	Balb/c mice	↓ Polychromatic erythrocyte (PCE) at 500 mg/kg of NSO, and NSO + Cisplatin which indicated a bone marrow recovery.	[72]
TQ (1, 10 and 25 µM)	In vitro	- Liver cancer cells SK-Hep 1 - Breast cancer cells MDA-MB 231	- Activation of cell apoptosis - ↑ P38 phosphorylation - ↑ Extracellular signal-regulated kinases (ERK). - Inhibition of cell proliferation at low doses of 50 and 100 µM	[73,74]
		Renal human cancer cells CaKi-1	- ↑ Pro-apoptotic markers P53, Bax, and Cytochrome C at 25 µM. - ↓ Anti-apoptotic markers Bcl-2 and Bcl-xl. - Inhibition of JAK2/STAT3 pathway phosphorylation which induces cyclin D1, cyclin D2, and the survivine inhibition. - ↑ ROS	
Ferulic acid (250, 350 and 450 µM)	In vitro	Breast cancer cells MDA-MB 231	- No effect was observed at 250 µM. - Antiproliferative action at a dose of 450 µM.	
TQ + ferulic acid (“25 µM + 250 µM”, “50 µM + 350 µM”, “50 µM + 450 µM”, “100 µM + 350 µM” and “100 µM + 450 (“25 µM + 250 µM” respectively)			25 µM + 250 µM antiproliferative effect	
Sapindoside B (1, 5, 10, 20 and 50 µM)	In vitro	HCT116, AGS, and on HCC-LM3	IC_50_ that was lower than 10 µM	[75]
		A549, H1299, H460, HGC27, and HepG2	IC_50_ ranging from 11.93 to 20.05 µM	
		Gastric cancer cells MGC830	Inactive	
*NS* virgin oil rich with volatile compounds (concentrations ranging from 0.46 to 3.09 mg/mL	In vitro	- MCF5 - A324	LC_50_ 1.6 µg/mL and 1.3 µg/mL for MCF5 and A325.	[76]
Virgin oil without volatile compounds			No effect even at high doses	
Combination of (*NS*, *Hemidesmus indicus*, and *smilax glabra*) ethyl acetate extract (25, 50, 100, 200 and 400 µg/mL)	In vitro	Lung cancer cells NCI-H292	- Cell morphology changes and reduction of the cell volume. - 300 µg/mL induced DNA fragmentation. - ↑ Caspase 3/7 activity at 50 µg/mL. - Subexpression of Bac and P53 induced apoptosis of cancer cells. - ↓ Heat shock proteins (Hsp) 70 and 90	[77]
Hydroalcoholic extract (100, 200, 400, 600 and 800 µg/mL)	In vitro	MCF-5	IC_50_ value noted was 3.29 mg/mL	[78]

↑: Upregulation; ↓: Downregulation.

**Table 4:** Summary of the anti-inflammatory and immunomodulatory activities of *NS.*

Extract/Compound	Method	Test type	Results	Reference
NSO (1, 2 and 4 mL/kg BW for the acute phase, 4 mL/kg BW for the subacute phase)	In vivo	Inflammation caused by carrageenan on the rat’s path	Suppression of the edema	[39,80]
		Acute treatment and subacute	- Doses of 2 and 4 mL/kg showed their anti-inflammatory in the acute test. - No activity in the subacute test.	
*NS* supplementation (200 mg/kg BW) (2 g/day added to foods or drinks)	In vivo	Injection of 10% PHA phytohemagglutinin to rats	- 50 g/kg daily supplementation induced an increase in the size of the spleen - ↑ IL-12. - ↑ TNF-α - ↑ IF-γ - ↑ CD8 production.	[81]
		Clinical study on patients with β-thalassemia	(2 g/d) for 3 months induced: - ↑ Neutrophils, ↑ CD4, and ↑ CD8 - ↑ Phagocytic capacity of macrophages	[82,83]
Hydroalcoholic extract	In vivo	* Oreochromis niloticus *	- ↓ NFK and ↓ IKK mRNAs - ↓ IL-1β - ↓ CYP1A	[49]
Ethanolic extract (1.2, 2.4 and 4.8 g/kg BW/day)	In vivo	On lupus mice	- ↓ anti-DNA - Improvement of the Th1, and Th2 balance - ↑ T-cells in lupus mice at 4.8 g/kg/d - 200 mg/kg which may balance the Th1/Th2 cytokine profile	[81,84]
*NS* polysaccharides (NSSP) (0.1 mL/10 g)	In vivo	Injection cyclophosphamide CTX to mice	Protection of thymus and spleen against CTX-induced damage - ↑ LDH - ↑ Acid phosphatase - ↑ TAC - ↑ SOD - ↑ CAT activity - ↓ MDA. - ↑ IL-2, IL-4, and IL-6 in the serum of mice. - ↓ TNF-α, and regulation of cytokines level.	[85]
TQ	In vitro	Human monocytic leukemia cell line THP-1	- Protection against subgingival inflammation - Inhibition of TNF-α	[48]

↑: Upregulation; ↓: Downregulation.

**Table 5:** Summary of the cardioprotective activities of *NS.*

Extract/Compound	Method	Test	Results	Reference
NSO (2.5 mL/day) (0.4 mL/100 g BW) (4 mL/kg BW/day) (400 mg/kg BW/day)	In vivo	Clinical on patients suffering from hypertension (8 weeks treatment)	↓ Systolic and Diastolic blood pressure	[86]
	In vivo	Pre-treatment of rats during 14 days by a dose of 4 mL/kg/day pursued with isoproterenol injection	- ↓ Troponin, ↓ Creatinine kinase - ↓ ASAT - No effect on the complex QRS or the PR interval - ↓ QT and QTc intervals - No effect on R-wave amplitude.	[30,87,88]
	In vivo	Cotreatment of rats using Azithromycin^®^ and NSO	- ↓ CPK - ↓ LDH - ↓ MDA - ↓TNFα - Preservation of cardiac morphology - ↓ Caspase-3.	
	In vivo	Female rats with streptozotocin-induced diabetes	- Normal histological structure after treatment with NSO - Hyperemia and hyaline degeneration in some muscle cells. - ↑ Bcl-2 overexpression	
*NS*supplementation (2000 mg/day)	In vivo	Clinical study on healthy obese and overweight subjects (2 g/day)	- ↓ Systolic pressure - No effect was observed on diastolic pressure	[89]
NS + amlodipine	In vivo	Hypertensive rats	- Better control of blood pressure - ↓ Heart rate	[90]
EtOH extract (400 mg/kg)	Ex vivo	Aortic ring	- ↓ Aortic contractions - Improvement of the reactivity of the aorta to vasoconstrictor and vasodilator agents	[91]
TQ (50 mg/kg BW/day) (10 mg/kg BW/day)	In vivo	Mice	↓ Lipid deposition at the cardiac tissue	[92,93]
	In vivo	Rats	- Two months treatment induced an inotropic effect characterized by maximal tension that is mediated by increased sensitivity of contractile proteins to Ca^2+^ - No effect on half-relaxation time and myocardial output	

↑: Upregulation; ↓: Downregulation.

**Table 6:** Summary of the antidiabetic activities of *NS.*

Extract/Fraction/Compound	Method	Test Type	Results	Reference
***NS* Seeds**				
*NS* supplementation (5 g/day) (1 g/day for 4 weeks) (1.5 mg/kg BW)	In vivo	Glycated hemoglobin (5 g/day of black cumin for 6 months)	↓ Glycated hemoglobin	[98]
		- Volunteers - Rats	- No effect on insulin secretion - Regeneration of exocrine and endocrine parts of pancreatic tissues	[105,110]
Hydroacetone extract (concentrations ranging between 156.25 and 2000 µg/mL)	In vitro	α-amylase	IC_50_ = 314.4 µg/mL	[97]
n-hexane fraction (0.45, 0.9 and 1.82 mg/mL)	In vitro	α -amylase	IC_50_ = 0.760 mg/mL	[19]
EtOH fraction (0.45, 0.9 and 1.82 mg/mL)			IC_50_ = 0.255 mg/mL	
MeOH fraction (0.45, 0.9 and 1.82 mg/mL)			IC_50_ = 0.103 mg/mL	
Aqueous fraction (0.45, 0.9 and 1.82 mg/mL)			IC_50_ = 0.310 mg/mL	
Dichloromethane fraction (0.45, 0.9 and 1.82 mg/mL)			IC_50_ = 1.330 mg/mL	
Hydroalcoholic combination (70%) of *Morinda citrifolia*, *Trigonella foenum-graecum,* and *NS* (15.625, 31.25, 62.5, 125 and 250 μg/mL) (3 doses: 70/70/280, 140/70/140 and 70/140/140 mg/kg BW)	In vitro	α -amylase	140/70/140 mg/kg was the most effective among the all doses with an IC_50_ = 35.7 µg/mL	[101]
	In vivo (rats)		↓ blood glucose level in rats	
n-hexane extract (400 mg/kg BW/day)	In vitro	- α -amylase - α -glucosidase - Glycated hemoglobin	- IC_50_ = 403.5 µg/mL - IC_50_ = 74.7 µg/mL - ↓ Glycated hemoglobin	[36,107]
Ethanolic extract (300 mg/kg BW/day) (1.5, 3.5 and 10 mg/mL)	In vivo	Rats with streptozotocin-induced diabetes	300 mg/kg of the *NS* induced: - ↓ Blood glucose (non-significant) - ↓ Fructosamine - Improvement in the number and morphology of islet cells	[103,106]
	In vitro	Glycation of bovine serum albumin	Antiglycation effect at different concentrations	
MeOH extract (Glucose tolerance/Residual gut sucrose content/Intestinal glucose absorption/Gut motility/Intestinal disaccharidase enzyme activity: 500 mg/kg BW) (Insulin secretion from isolated islets: 25, 50, 100 and 200 μg/mL)	In vivo	Long–Evans rats	- ↓ Blood glucose - Suppression of sucrose-induced hyperglycemia - ↑ unabsorbed sucrose - Inhibition of disaccharidase enzyme	[104]
Aqueous extract (1.5, 3.5 and 10 mg/mL)	In vitro	Glycation of bovine serum albumin	Antiglycation effect at different concentrations	[106]
*Trigonella foenum-graecum*, *NS*, *Zingiber officinale*, *Olea europea*, *Fraxinus* ssp (water 80%–MeOH 20%) (concentrations of extracts graded from 10–100 mg/kg BW)	In vivo	Rats	- Protection of the pancreas’ proper functioning - Ultrastructure study by transmission electron microscope (TEM) showed that the acinar cells had normal ultrastructure with normal cell activity and nuclei, and well-developed rough endoplasmic reticulum with abundant zymogen granules - Protection against the degeneration caused by diabetes induced by alloxan injection	[109]
*NS* + fenugreek (4.7 and 0.75 g powdered NS and fenugreek respectively seed/day)	In vivo	Clinical study on overweight, type 2 diabetes, and overweight + type 2 diabetes	- ↓ Glycated hemoglobin - ↓ Liver enzymes	[102]
TQ (50 or 100 mg) with metformin (1000 mg)	In vivo	Clinical study on volunteers	- Adverse effects in some of the volunteers such as diarrhea and abdominal pain - ↓ Glycated hemoglobin was observed after 3 months - ↓ Fasting blood glucose - ↓ Postprandial blood glucose	[100]
**Aerial part**				
Hederagenin,	In vitro (12.5, 25, 50 and 100 µM)	- α-glucosidase - Phosphatase tyrosine kinase (PTP1B)	- IC_50_ ranging from 256.7 ±3.7 µM to 331.9 ± 1.6 µM - No activity on PTP1B	[20]
Flaccidoside III,				
Quercetin-3-gentiobiosides,				
Magnoflorin,				
Nigelflavonoside B,				
Nigelloside,				
Quercetin sphorotrioside,				
Kaempferol-3, 7-diglucoside,				
Kaempferol 3-O- rutinoside				
Rutin				
3-O-[α-L-Rhamnopyranosyl-(1-2)-α-l-arabinopyranpsyl] hederagenin		- Phosphatase tyrosine kinase (PTP1B)	IC_50_ of 91.3 ± 2.5µM	

↑: Upregulation; ↓: Downregulation.

**Table 7:** Summary of the hypolipidemic activities of *NS.*

Extract/Compound	Method	Test Type	Results	Reference
***NS* Seeds**				
NSO (0.4 mL/100 g BW)	In vivo	Rats	- ↓ Total cholesterol - ↓ Low-density lipoproteins (LDL) - ↓ Malondialdehyde (MDA) - ↑ High-density lipoproteins (HDL) - ↑ Glutathione reductase	[30]
*NS* supplementation (2 g/day)	In vivo	Obese and overweight healthy women	- ↑ HDL. - ↓ Atherogenicity index - ↓ LDL - ↓ Serum glutamic-oxaloacetic transaminase	[89]
*NS*+ fenugreek (4.7:0.75 g/day respectively)	In vivo	overweight, type 2 diabetes, and overweight + type 2 diabetes volunteers	- ↓ LDL - ↓ TG - ↓ VLDL - ↓ Atherogenicity index.	[102]
TQ (50 mg/kg BW/day	In vivo	Mice	- ↓ Total cholesterol - ↓ LDL - ↓ C-reactive proteins	[92]
Hydroalcoholic extract (100, 200, and 400 mg/kg)	In vivo	Rats	Six weeks treatment - ↓cholesterol - ↓ TG - ↓ LDL - ↑ HDL	[111]
*NS* + fenugreek (2 g:10 g/d)	In vivo	Volunteer patients with type 2 diabetes	- ↓ Cholesterol - ↓ LDL - ↓ MDA - ↓ Triglycerides - ↑ HDL	[112]
*Artemisia sieberi*, *NS,* and *Teucrium polium* (150 mg/kg BW)	In vivo	Rats	- ↓ Cholesterol - ↓ LDL - ↓ Triglycerides - ↓ ALAT and ASAT - ↑ HDL	[108]

↑: Upregulation; ↓: Downregulation.

## Data Availability

Not applicable.

## Abbreviation

*NS*: *Nigella sativa*; NSO, *Nigella sativa* oil; TQ, Thymoquinone; DPPH, 1,1-diphenyl-2-picryl hydrazine; MDA, Malondialdehyde; GSH, reduced glutathione; HUVEC, Human umbilical vein endothelial cells; H_2_O_2_, Hydrogen peroxide; DNA, Deoxyribonucleic acid; UVA, Ultraviolet A-rays; HaCaT, human keratinocytes cells; COX-2, Cyclooxygenase-2; IC50, The half maximal inhibitory concentration; ABTS, 2,2-azino-bis(3-ethylbenzothiazoline-6-sulfonic acid; CAT, Catalase; SOD, Superoxide dismutase; FRAP, Ferric Reducing Power; STZ, Streptozotocin; GSSG, Oxidized Glutathione; MDR, Multi-drug resistance; ATB, Antibiotics; MRSA, Methicillin resistant *Staphylococcus aureus; ESBL, extended spectrum beta-lactamase;* EOs, Essential Oils; CYP1A, Cytochrome P450 1 A; ACE2, angiotensin-converting enzyme 2; HCQ, hydroxychloroquine; RNA, Ribonucleic acid; TMPRSS2, Transmembrane serine protease 2; PA-1, human ovarian teratocarcinoma cell line; B16 melanoma, murine tumor cell line; HTC-116, Human adenocarcinoma; HPF, Human postnatal fibroblasts; SK-Hep 1, Liver cancer cells; MAPK, Mitogen-Activated Protein Kinases; ERK, extracellular signal-regulated kinases; MDA-MB 231, Breast cancer cells; THP-1, Human monocytic cell line; HEK293, Embryonic kidney cells; TLR4, Toll Like Receptor 4; SW620, Colorectal cancer metastasis; JNK, c-Jun N-terminal kinases; PCE, polychromatic erythrocyte; CaKi-1, Renal human cancer cells; ROS, Reactive oxygen species; NCI-H292, Lungs cancer cells; Hsp, Heat Shock Protein; PHA, Phytohemaglutinin; IL-2, Interleukin 2; TNF-α, Tumor necrosis factor; IFγ, Interferon; IKK, IKB kinase; IL-1β, Interleukin 1 β; NSSP, *Nigella sativa* polysaccharides; CTX, Cyclophosphamide; IL-4, Interleukin 4; IL-6, Interleukin 6; PTEN, phosphatase and TENsin homolog; SLE, Systemic lupus erythematosus; Th1, T helper 1; Th2, T helper 2; CD8, cluster of differentiation 8; CD4, cluster of differentiation 4; ISO, isoproterenol; ASAT, Aspartate aminotransferase; ALAT, Alanine aminotransferase; GH, Growth hormone; CPK, Creatinine phosphokinase; LDH, lactate dehydrogenase; α-SMA, Alpha smooth muscle actine; L-NAME, N(gamma)-nitro-L-arginine methyl ester; SBP, Systolic blood pressure; DBP, Diastolic blood pressure, MAP, Mean arterial blood pressure; HbA1c, glycated hemoglobin; MeOH, Methanol; EtOH, Ethanol; Hex, hexane; PTP1B, phosphatase tyrosine kinase; TEM, transmission electron microscope; TG, triglycerides; LDL, low-density lipoproteins; HDL, High-density lipoproteins; vLDL, Very low-density lipoproteins; VCAM1, vascular cell adhesion molecule 1; PI3K, Phosphatidylinositol 3-kinases; NF-B, Nuclear factor-kappa B; Akt, Protein kinase B; MGC830, Gastric cancer cells.

## References

[1] Gharby S., Harhar H., Guillaume D., Roudani A., Boulbaroud S., Ibrahimi M., Ahmad M., Sultana S., Hadda T.B., Chafchaouni-Moussaoui I. (2015). Chemical investigation of *Nigella sativa* L. seed oil produced in Morocco. J. Saudi Soc. Agric. Sci..

[2] Shabana A., El-Menyar A., Asim M., Al-Azzeh H., Al Thani H. (2013). Cardiovascular benefits of black cumin (*Nigella sativa*). Cardiovasc. Toxicol..

[3] Kehili N., Saka S., Aouacheri O. (2018). L’effet phytoprotecteur de la nigelle (*Nigella sativa*) contre la toxicité induite par le cadmium chez les rats. Phytothérapie.

[4] Medhi H. (2019). Contribution à l’étude de la graine de nigelle ou cumin noir *Nigella sativa* L.. Master’s Thesis.

[5] Ghedira K. (2006). La nigelle cultivée: *Nigella sativa* L. (Ranunculaceae). Phytotherapie.

[6] Fakchich J., Elachouri M. (2021). An overview on ethnobotanico-pharmacological studies carried out in Morocco, from 1991 to 2015: Systematic review (part 1). J. Ethnopharmacol..

[7] Dalli M., Azizi S., Kandsi F., Gseyra N. (2021). Evaluation of the in vitro antioxidant activity of different extracts of *Nigella sativa* L. seeds, and the quantification of their bioactive compounds. Mater. Today Proc..

[8] Dalli M., Azizi S., Benouda H., Azghar H.A., Tahri M., Boufalja B., Maleb A., Gseyra N. (2021). Molecular Composition and Antibacterial Effect of Five Essential Oils Extracted from *Nigella sativa* L. Seeds against Multidrug-Resistant Bacteria: A Comparative Study. Evid.-Based Complement. Altern. Med..

[9] Shahid M.A., Rahim A., Chowdhury M.A., Kashem M.A. (2021). Development of antibacterial nanofibrous wound dressing and conceptual reaction mechanism to deactivate the viral protein by *Nigella sativa* extract. Adv. Tradit. Med..

[10] Hwang J.R., Cartron A.M., Khachemoune A. (2021). A review of *Nigella sativa* plant-based therapy in dermatology. Int. J. Dermatol..

[11] Alhmied F., Alammar A., Alsultan B., Alshehri M., Pottoo F.H. (2021). Molecular Mechanisms of Thymoquinone as Anticancer Agent. Comb. Chem. High Throughput Screen..

[12] Hossain M.S., Sharfaraz A., Dutta A., Ahsan A., Masud M.A., Ahmed I.A., Goh B.H., Urbi Z., Sarker M.M.R., Ming L.C. (2021). A review of ethnobotany, phytochemistry, antimicrobial pharmacology and toxicology of *Nigella sativa* L.. Biomed. Pharmacother..

[13] Majdalawieh A.F., Fayyad M.W. (2016). Recent advances on the anti-cancer properties of *Nigella sativa*, a widely used food additive. J. Ayurveda Integr. Med..

[14] Kulyar M.F.-E.-A., Li R., Mehmood K., Waqas M., Li K., Li J. (2021). Potential influence of Nagella sativa (Black cumin) in reinforcing immune system: A hope to decelerate the COVID-19 pandemic. Phytomedicine.

[15] Ansary J., Giampieri F., Forbes-Hernandez T.Y., Regolo L., Quinzi D., Gracia Villar S., Garcia Villena E., Tutusaus Pifarre K., Alvarez-Suarez J.M., Battino M. (2021). Nutritional Value and Preventive Role of *Nigella sativa* L. and Its Main Component Thymoquinone in Cancer: An Evidenced-Based Review of Preclinical and Clinical Studies. Molecules.

[16] Hannan M., Rahman M., Sohag A.A.M., Uddin M., Dash R., Sikder M.H., Timalsina B., Munni Y.A., Sarker P.P., Alam M. (2021). Black cumin (*Nigella sativa* L.): A comprehensive review on phytochemistry, health benefits, molecular pharmacology, and safety. Nutrients.

[17] Kabir Y., Akasaka-Hashimoto Y., Kubota K., Komai M. (2020). Volatile compounds of black cumin (*Nigella sativa* L.) seeds cultivated in Bangladesh and India. Heliyon.

[18] Kumar S.P.P.K. (2017). Advances in Biochemistry of Medicinal Plants. Biochemistry and Therapeutic Uses of Medicinal Plants.

[19] Dalli M., Daoudi N.E., Azizi S., Benouda H., Bnouham M., Gseyra N. (2021). Chemical Composition Analysis Using HPLC-UV/GC-MS and Inhibitory Activity of Different *Nigella sativa* Fractions on Pancreatic α-Amylase and Intestinal Glucose Absorption. BioMed Res. Int..

[20] Parveen A., Farooq M.A., Kyunn W.W. (2020). A new oleanane type saponin from the aerial parts of nigella sativa with anti-oxidant and anti-diabetic potential. Molecules.

[21] Atta-ur-Rahman S.M. (1985). Isolation and structure determination of nigellicine, a novel alkaloid from the seeds of nigella sativa. Tetrahedron Lett..

[22] Atta-ur-Rahman S.M., Zaman K. (1992). Nigellimine: A new isoquinoline alkaloid from the seeds of nigella sativa. J. Nat. Prod..

[23] Atta-ur-Rahman S.M., Hasan S.S., Choudhary M.I., Ni C.Z., Clardy J. (1995). Nigellidine—A new indazole alkaloid from the seeds of *Nigella sativa*. Tetrahedron Lett..

[24] Makkar H.P.S., Siddhuraju P., Becker K. (2007). Plant Secondary Metabolites.

[25] Taşkin M.K., Çalişkan Ö.A., Anil H., Abou-Gazar H., Khan I.A., Bedir E. (2005). Triterpene saponins from *Nigella sativa* L.. Turk. J. Chem..

[26] Takruri H.R.H., Dameh M.A.F. (1998). Study of the nutritional value of black cumin seeds (*Nigella sativa* L). J. Sci. Food Agric..

[27] Tiji S., Benayad O., Berrabah M., El Mounsi I., Mimouni M. (2021). Phytochemical Profile and Antioxidant Activity of *Nigella sativa* L. Growing in Morocco. Sci. World J..

[28] Nickavar F., Mojab B., Javidnia K., Amoli Roodgar M.A. (2003). Chemical Composition of the Fixed and Volatile Oils of *Nigella sativa* L. from Iran. Z. Naturforsch.-Sect. C J. Biosci..

[29] Mahmoud H.S., Almallah A.A., L-Hak H.N.G.E., Aldayel T.S., Abdelrazek H.M.A., Khaled H.E. (2021). The effect of dietary supplementation with *Nigella sativa* (black seeds) mediates immunological function in male Wistar rats. Sci. Rep..

[30] Bocsan V.S., Pop I.C., Sabin R.M., Sarkandy O., Boarescu E., Roşian P.M., Leru Ş.H., Chedea P.M., Socaci S.A., Buzoianu A.D. (2021). Comparative protective effect of nigella sativa oil and vitis vinifera seed oil in an experimental model of isoproterenol-induced acute myocardial ischemia in rats. Molecules.

[31] Bilto Y.Y., Alabdallat N.G., Atoom A.M., Khalaf N.A. (2021). Effects of commonly used medicinal herbs in Jordan on erythrocyte oxidative stress oxidative. J. Pharm. Pharmacogn. Res..

[32] Farshori A.A., Saquib N.N., Siddiqui Q., Al-Oqail M.A., Al-Sheddi M.M., Al-Massarani E.S., Al-Khedhairy S.M. (2021). Protective effects of *Nigella sativa* extract against H2O2-induced cell death through the inhibition of DNA damage and cell cycle arrest in human umbilical vein endothelial cells (HUVECs). J. Appl. Toxicol..

[33] Liang J., Lian L., Wang X., Li L. (2021). Thymoquinone, extract from *Nigella sativa* seeds, protects human skin keratinocytes against UVA-irradiated oxidative stress, inflammation and mitochondrial dysfunction. Mol. Immunol..

[34] Rathi B., Devanesan S., AlSalhi M.S., Singh A.J.R. (2021). In-vitro free radical scavenging effect and cytotoxic analysis of Black Cummins and Honey formulation. Saudi J. Biol. Sci..

[35] Adebayo-Tayo B.C., Briggs-Kamara A.I., Salaam A.M. (2021). Phytochemical composition, antioxidant, antimicrobial potential and gc-ms analysis of crude and partitioned fractions of *Nigella sativa* seed extract. Acta Microbiol. Bulg..

[36] Bonesi R., Saab M., Tenuta A.M., Leporini M.C., Saab M., Loizzo M.J., Tundis M.R. (2020). Screening of traditional Lebanese medicinal plants as antioxidants and inhibitors of key enzymes linked to type 2 diabetes. Plant Biosyst..

[37] Babar Z.M., Azizi W.M., Ichwan S.J.A., Ahmed Q.U., Azad A.K., Mawa I. (2019). A simple method for extracting both active oily and water soluble extract (WSE) from *Nigella sativa* (L.) seeds using a single solvent system. Nat. Prod. Res..

[38] Jan K., Ahmad M., Rehman S., Gani A., Khaqan K. (2019). Effect of roasting on physicochemical and antioxidant properties of kalonji (*Nigella sativa*) seed flour. J. Food Meas. Charact..

[39] Vesa S.C., Chedea V.S., Bocsan I.C., Ancut S., Buzoianu A.D. (2020). *Nigella sativa*’s Anti-Inflammatory and Antioxidative Effects in Experimental Inflammation. Antioxidants.

[40] Nehar S., Rani P., Kumar C. (2021). Evaluation of genoprotective and antioxidative potentiality of ethanolic extract of N. sativa seed in streptozotocin induced diabetic albino rats. Vegetos.

[41] Alkis H., Demir E., Taysi M.R., Sagir S., Taysi S. (2021). Effects of *Nigella sativa* oil and thymoquinone on radiation-induced oxidative stress in kidney tissue of rats. Biomed. Pharmacother..

[42] Badger-Emeka L.I., Emeka P.M., Ibrahim H.I.M. (2021). A Molecular Insight into the Synergistic Mechanism of *Nigella sativa* (Black Cumin) with Β-Lactam Antibiotics against Clinical Isolates of Methicillin-Resistant Staphylococcus aureus. Appl. Sci..

[43] Arif P.L., Saqib S., Mubashir H., Malik M., Mukhtar S.I., Saqib A., Ullah S., Show S. (2021). Comparison of *Nigella sativa* and Trachyspermum ammi via experimental investigation and biotechnological potential. Chem. Eng. Process.-Process Intensif..

[44] Alizadeh-naini M., Yousefnejad H., Hejazi N. (2020). The beneficial health effects of *Nigella sativa* on Helicobacter pylori eradication, dyspepsia symptoms, and quality of life in infected patients: A pilot study. Phyther. Res..

[45] Raveesha K.A. (2021). Raveesha Antibacterial activity and time-kill assay of Terminalia catappa L. And *Nigella sativa* L. And selected human pathogenic bacteria. J. Pure Appl. Microbiol..

[46] Dera A.A., Ahmad I., Rajagopalan P., Al Shahrani M., Saif A., Alshahrani M.Y., Alraey Y., Alamri A.M., Alasmari S., Makkawi M. (2021). Synergistic efficacies of thymoquinone and standard antibiotics against multi-drug resistant isolates. Saudi Med. J..

[47] Habib N., Choudhry S. (2021). HPLC Quantification of Thymoquinone Extracted from *Nigella sativa* L. (Ranunculaceae) Seeds and Antibacterial Activity of Its Extracts against Bacillus Species. Evid.-Based Complement. Altern. Med..

[48] Tada T., Nakayama-Imaohji A., Yamasaki H., Elahi H., Nagao M., Yagi T., Ishikawa H., Shibuya M., Kuwahara K. (2020). Effect of thymoquinone on Fusobacterium nucleatum-associated biofilm and inflammation. Mol. Med. Rep..

[49] Hal A.M., El-Barbary M.I. (2021). Effect of *Nigella sativa* oil and ciprofloxacin against bacterial infection on gene expression in Nile tilapia (Oreochromis niloticus) blood. Aquaculture.

[50] Widdatallah M.O., Mohamed R., Alrasheid A.A., Widatallah A.A., Yassin H.A., Eltilib L.F., Abdel S.H., Ahmed S. (2020). Green Synthesis of Silver Nanoparticles Using *Nigella sativa* Seeds and Evaluation of Their Antibacterial Activity. Adv. Nanopart..

[51] Nazarparvar M., Shakeri A., Ranjbariyan A. (2020). Chemical composition and antimicrobial activity against food poisoning of alcoholic extract of *Nigella sativa* L.. Biointerface Res. Appl. Chem..

[52] Yasmin S., Nawaz K., Anjum M., Ashraf A.A., Basra I., Mehmood M.A.R., Khan A., Malik F. (2020). Phytochemical analysis and in vitro activity of essential oils of selected plants against Salmonella enteritidis and Salmonella gallinarum of poultry origin. Pak. Vet. J..

[53] Mosolygó G., Mouwakeh T., Ali A., Kincses M.H., Mohácsi-Farkas A., Kiskó C., Spengler G. (2019). Bioactive compounds of *Nigella sativa* essential oil as antibacterial agents against Chlamydia trachomatis D. Microorganisms.

[54] Nayef Y.A., Zangana B.S. (2020). Effect of essential oils on chemical composition and microbial load of minced and frozen stored chicken meat. Biochem. Cell. Arch..

[55] Hetta H.F., Meshaal A.K., Algammal A.M., Yahia R., Makharita R.R., Marraiki N., Shah M.A., Hassan H.A.M., Batiha G.E.S. (2020). In-vitro antimicrobial activity of essential oils and spices powder of some medicinal plants against bacillus species isolated from raw and processed meat. Infect. Drug Resist..

[56] Barashkova A.S., Sadykova V.S., Salo V.A., Zavriev S.K., Rogozhin E.A. (2021). Nigellothionins from black cumin (*Nigella sativa* l.) seeds demonstrate strong antifungal and cytotoxic activity. Antibiotics.

[57] Pournajafian M., Naseri A., Fata A., Rakhshandeh A., Afzal-Aghaee H. (2021). The antifungal effects of hydroalcoholic extracts of *Nigella sativa* and urtica dioica on fungal agents in comparison with amphotericin B. J. Isfahan Med. Sch..

[58] Rusda M., Adenin I., Siregar M.F.G., Rambe A.Y.M., Sudewo Y. (2021). Therapeutic effect of 48 h after *Nigella sativa* extract administration on female wistar rats vaginal candidiasis model: An experimental study. Open Access Maced. J. Med. Sci..

[59] Aftab A., Yousaf Y., Javaid A., Riaz N., Younas A., Rashid M., Shamsher B., Arif A. (2019). Antifungal activity of vegetative methanolic extracts of *Nigella sativa* against Fusarium oxysporum and Macrophomina phaseolina and its phytochemical profiling by GC-MS analysis. Int. J. Agric. Biol..

[60] Koshak A.E., Koshak E.A., Mobeireek A.F., Badawi M.A., Wali S.O., Malibary H.M., Atwah A.F., Alhamdan M.M., Almalki R.A., Madani T.A. (2021). Complementary Therapies in Medicine *Nigella sativa* for the treatment of COVID-19: An open-label randomized controlled clinical trial. Complement. Ther. Med..

[61] Kadil Y., Mouhcine M., Filali H. (2021). In Silico Investigation of the SARS-CoV2 Protease with Thymoquinone, the Major Constituent of *Nigella sativa*. Curr. Drug Discov. Technol..

[62] Sommer A.P., Försterling H.D., Sommer K.E. (2021). Tutankhamun’s Antimalarial Drug for COVID-19. Drug Res..

[63] Khan S.A. (2021). Combating COVID-19: The role of drug repurposing and medicinal plants. J. Infect. Public Health.

[64] Mir S.A., Firoz A., Alaidarous M., Alshehri B., Dukhyil A.A.B., Banawas S., Alsagaby S.A., Alturaiki W., Bhat G.A., Kashoo F. (2021). Identification of SARS-CoV-2 RNA-dependent RNA polymerase inhibitors from the major phytochemicals of *Nigella sativa*: An in silico approach. Saudi J. Biol. Sci..

[65] Yadav P.K., Jaiswal A., Singh R.K. (2021). In silico study on spice-derived antiviral phytochemicals against SARS-CoV-2 TMPRSS2 target. J. Biomol. Struct. Dyn..

[66] Ahmad S., Abbasi H.W., Shahid S., Gul S., Abbasi S.W. (2021). Molecular docking, simulation and MM-PBSA studies of nigella sativa compounds: A computational quest to identify potential natural antiviral for COVID-19 treatment. J. Biomol. Struct. Dyn..

[67] Sumaryada T., Pramudita C.A. (2021). Molecular docking evaluation of some indonesian’s popular herbals for a possible COVID-19 treatment. Biointerface Res. Appl. Chem..

[68] El-Sayed S.A.E.S., Rizk M.A., Yokoyama N., Igarashi I. (2019). Evaluation of the in vitro and in vivo inhibitory effect of thymoquinone on piroplasm parasites. Parasites Vectors.

[69] Dagtas A.S., Griffin R.J. (2021). *Nigella sativa* extract kills pre-malignant and malignant oral squamous cell carcinoma cells. J. Herb. Med..

[70] El-Obeid A., Alajmi H., Harbi M., Yahya W.B., Al-Eidi H., Alaujan M., Haseeb A., Trivilegio T., Alhallaj A., Alghamdi S. (2020). Distinct anti-proliferative effects of herbal melanin on human acute monocytic leukemia thp-1 cells and embryonic kidney hek293 cells. BMC Complement. Med. Ther..

[71] Al-Obeed O., El-Obeid A.S., Matou-Nasri S., Vaali-Mohammed M.A., AlHaidan Y., Elwatidy M., Al Dosary H., Alehaideb Z., Alkhayal K., Haseeb A. (2020). Herbal melanin inhibits colorectal cancer cell proliferation by altering redox balance, inducing apoptosis, and modulating MAPK signaling. Cancer Cell Int..

[72] Franco-Ramos R.S., López-Romero C.A., Torres-Ortega H., Oseguera-Herrera D., Lamoreaux-Aguayo J.P., Molina-Noyola D., Juárez-Vázquez C.I., Torres-Bugarín O. (2020). Evaluation of anti-cytotoxic and anti-genotoxic effects of *Nigella sativa* through a micronucleus test in balb/c mice. Nutrients.

[73] Al-Mutairi A., Rahman A., Rao M.S. (2021). Low Doses of Thymoquinone and Ferulic Acid in Combination Effectively Inhibit Proliferation of Cultured MDA-MB 231 Breast Adenocarcinoma Cells. Nutr. Cancer.

[74] Chae I.G., Song N.Y., Kim D.H., Lee M.Y., Park J.M., Chun K.S. (2020). Thymoquinone induces apoptosis of human renal carcinoma Caki-1 cells by inhibiting JAK2/STAT3 through pro-oxidant effect. Food Chem. Toxicol..

[75] Hu X., Lin M., Zhu W., Zheng Y., Zhang Q., Wu G., Qiu Y. (2020). Potential Cytotoxicity, Pharmacokinetics, and Excretion Properties of Sapindoside B from the Seeds of *Nigella sativa* var hispidula. Planta Med..

[76] Silva A.F.C., Haris P.I., Serralheiro M.L., Pacheco R. (2020). Mechanism of action and the biological activities of *Nigella sativa* oil components. Food Biosci..

[77] Pathiranage V.C., Thabrew I., Samarakoon S.R., Tennekoon K.H., Rajagopalan U., Ediriweera M.K. (2020). Evaluation of anticancer effects of a pharmaceutically viable extract of a traditional polyherbal mixture against non-small-cell lung cancer cells. J. Integr. Med..

[78] Kordestani Z., Shahrokhi-Farjah M., Rouholamini S.E.Y., Saberi A. (2020). Reduced ikk/nf-kb expression by *Nigella sativa* extract in breast cancer. Middle East J. Cancer.

[79] Zhang B., Ting W.J., Gao J., Kang Z.F., Huang C.Y., Weng Y.J. (2021). Erk phosphorylation reduces the thymoquinone toxicity in human hepatocarcinoma. Environ. Toxicol..

[80] Mutabagani A., El-Mahdy S.A. (1997). A study of the anti-inflammatory activity of *Nigella sativa* L. and thymoquinone in rats. Saudi Pharm. J..

[81] Gholamnezhad Z., Boskabady M.H., Hosseini M. (2021). The effect of chronic supplementation of *Nigella sativa* on splenocytes response in rats following treadmill exercise. Drug Chem. Toxicol..

[82] El-Shanshory M., Hablas N.M., Aboonq M.S., Fakhreldin A.R., Attia M., Arafa W., Mariah R.A., Baghdadi H., Ayat M., Zolaly M. (2019). *Nigella sativa* improves anemia, enhances immunity and relieves iron overload-induced oxidative stress as a novel promising treatment in children having beta-thalassemia major. J. Herb. Med..

[83] Hakim A.S., Abouelhag H.A., Abdou A.M., Fouad E.A., Khalaf D.D. (2019). Assessment of Immunomodulatory Effects of Black Cumin Seed (*Nigella sativa*) Extract on Macrophage Activity in Vitro. Int. J. Vet. Sci..

[84] Guritno T., Barlianto W., Wulandari D., Amru W.A. (2021). Effect *Nigella sativa* extract for balancing immune response in pristane induced lupus mice model. J. Appl. Pharm. Sci..

[85] Liang Q., Dong J., Wang S., Shao W., Ahmed A.F., Zhang Y., Kang W. (2021). Immunomodulatory effects of *Nigella sativa* seed polysaccharides by gut microbial and proteomic technologies. Int. J. Biol. Macromol..

[86] Shoaei-Hagh P., Kamelan Kafi F., Najafi S., Zamanzadeh M., Heidari Bakavoli A., Ramezani J., Soltanian S., Asili J., Hosseinzadeh H., Eslami S. (2021). A randomized, double-blind, placebo-controlled, clinical trial to evaluate the benefits of *Nigella sativa* seeds oil in reducing cardiovascular risks in hypertensive patients. Phytother. Res..

[87] El-Kader M.A. (2020). Evaluation of azithromycin induced cardiotoxicity in male albino rats and the possible protective role of *Nigella sativa* oil. Egypt. J. Histol..

[88] Altun E., Avci E., Yildirim T., Yildirim S. (2019). Protective effect of *Nigella sativa* oil on myocardium in streptozotocin-induced diabetic rats. Acta Endocrinol..

[89] Razmpoosh E., Safi S., Nadjarzadeh A., Fallahzadeh H., Abdollahi N., Mazaheri M., Nazari M., Salehi-Abargouei A. (2021). The effect of *Nigella sativa* supplementation on cardiovascular risk factors in obese and overweight women: A crossover, double-blind, placebo-controlled randomized clinical trial. Eur. J. Nutr..

[90] Alam M.A., Jardan Y.A.B., Raish M., Al-Mohizea A.M., Ahad A., Al-Jenoobi F.I. (2020). Effect of *Nigella sativa* and Fenugreek on the Pharmacokinetics and Pharmacodynamics of Amlodipine in Hypertensive Rats. Curr. Drug Metab..

[91] Hosseinzadeh H., Tafaghodi M., Mosavi M.J., Taghiabadi E. (2013). Effect of Aqueous and Ethanolic Extracts of *Nigella sativa* Seeds on Milk Production in Rats. JAMS J. Acupunct. Meridian Stud..

[92] Pei Z.W., Guo Y., Zhu H.L., Dong M., Zhang Q., Wang F. (2020). Thymoquinone Protects against Hyperlipidemia-Induced Cardiac Damage in Low-Density Lipoprotein Receptor-Deficient (LDL-R-/-) Mice via Its Anti-inflammatory and Antipyroptotic Effects. BioMed Res. Int..

[93] Al Asoom L.I., Al-Hariri M.T. (2019). Cardiac Inotropic Effect of Long-Term Administration of Oral Thymoquinone. Evid.-Based Complement. Altern. Med..

[94] Al Asoom L.I. (2021). Molecular Mechanisms of *Nigella sativa*—And *Nigella sativa* Exercise-Induced Cardiac Hypertrophy in Rats. Evid.-Based Complement. Altern. Med..

[95] Mousavi S.M., Abbasnezhad A., Mohebbati R., Kianmehr M., Ghorbani M. (2020). Comparative effects of Glibenclamide and *Nigella sativa* on aortic contractile and dilation response in diabetic rat. Acta Med. Mediterr..

[96] Jardan Y.A.B., Ahad A., Raish M., Alam M.A., Al-Mohizea A.M., Al-Jenoobi F.I. (2021). Effects of garden cress, fenugreek and black seed on the pharmacodynamics of metoprolol: An herb-drug interaction study in rats with hypertension. Pharm. Biol..

[97] Varghese L.N., Mehrotra N. (2020). α-Amylase inhibitory activity of microencapsulated *Nigella sativa* L. and herb- drug interaction: An in vitro analysis. Ann. Phytomed. Int. J..

[98] Hamdan A., Haji Idrus R., Mokhtar M.H. (2020). Effects of *Nigella sativa* on type-2 diabetes mellitus: A systematic review. Int. J. Environ. Res. Public Health.

[99] Vijayakumar S., Divya M., Vaseeharan B., Chen J., Biruntha M., Silva L.P., Durán-Lara E.F., Shreema K., Ranjan S., Dasgupta N. (2021). Biological Compound Capping of Silver Nanoparticle with the Seed Extracts of Blackcumin (*Nigella sativa*): A Potential Antibacterial, Antidiabetic, Anti-inflammatory, and Antioxidant. J. Inorg. Organomet. Polym. Mater..

[100] Ali S.M., Chen P., Sheikh S., Ahmad A., Ahmad M., Paithankar M., Desai B., Patel P., Khan M., Chaturvedi A. (2021). Thymoquinone with Metformin Decreases Fasting, Post Prandial Glucose, and HbA1c in Type 2 Diabetic Patients. Drug Res..

[101] Ida M., Ahmad M., Dwi A.N. (2020). Fruit, Trigonella foenum -graecum and *Nigella sativa* L. Seeds Using In vitro and In vivo Assay. Trop. J. Nat. Prod. Res..

[102] Rao A.S., Hegde S., Pacioretty L.M., Debenedetto J., Babish J.G. (2020). *Nigella sativa* and Trigonella foenum-graecum Supplemented Chapatis Safely Improve HbA1c, Body Weight, Waist Circumference, Blood Lipids, and Fatty Liver in Overweight and Diabetic Subjects: A Twelve-Week Safety and Efficacy Study. J. Med. Food.

[103] Ibrahim H.A.E., Hashem M.A., Mohamed N.E., El-Rahman A.A.A. (2020). Assessment of ameliorative effects of zingiber officinale and *Nigella sativa* on streptozotocin-induced diabetic rats. Adv. Anim. Vet. Sci..

[104] Hannan J.M.A., Ansari P., Haque A., Sanju A., Huzaifa A., Rahman A., Ghosh A., Azam S. (2019). *Nigella sativa* stimulates insulin secretion from isolated rat islets and inhibits the digestion and absorption of (CH_2_O)n in the gut. Biosci. Rep..

[105] Pelegrin S., Galtier F., Chalançon A., Gagnol J.P., Barbanel A.M., Pélissier Y., Larroque M., Lepape S., Faucanié M., Gabillaud I. (2019). Effects of *Nigella sativa* seeds (black cumin) on insulin secretion and lipid profile: A pilot study in healthy volunteers. Br. J. Clin. Pharmacol..

[106] Ramdan B., Ramdan R., El Karbane M., El Maadoudi M., Ben Mrid R., Nhiri M. (2019). Anti-glycation study of hydro-alcohol and aqueous extracts of Moroccan plant species. Int. J. Res. Pharm. Sci..

[107] Bonab S.B., Tofighi A. (2019). Effect of 8 weeks aerobic training and nigella supplement on insulin resistance, lipid profile and plasma level of hba1c in type 2 diabetic rats. J. Adv. Med. Biomed. Res..

[108] Muhsin S.M., Mahmood R.I., Abdul-Lattif R.F., Sabrei D.A. (2019). Hypoglycemic and hypolipidemic properties of three plants extract in Alloxan induced diabetic rats. Plant Arch..

[109] Hadi M.A. (2019). E4An ultrastructural study by transmission electron microscope of exocrine pancreatic cells in diabetic rats treated with herbal combination. Baghdad Sci. J..

[110] Aboul-Mahasen L.M., Alshali R.A. (2019). The possible protective effects of virgin olive oil and *Nigella sativa* seeds on the biochemical and histopathological changes in pancreas of hyperlipidaemic rats. Folia Morphol..

[111] Abbasnezhad A., Niazmand S., Mahmoudabady M., Rezaee S.A., Soukhtanloo M., Mosallanejad R., Hayatdavoudi P. (2019). *Nigella sativa* L. seed regulated eNOS, VCAM-1 and LOX-1 genes expression and improved vasoreactivity in aorta of diabetic rat. J. Ethnopharmacol..

[112] Shari F.H., Ramadhan H.H., Mohammed R.N., Al-Bahadily D.C. (2020). Hypolipidemic and antioxidant effects of fenugreek- *Nigella sativa* combination on diabetic patients in Iraq. Syst. Rev. Pharm..

[113] Zaoui A., Cherrah Y., Mahassini N., Alaoui K., Amarouch H., Hassar M. (2002). Acute and chronic toxicity of *Nigella sativa* fixed oil. Phytomedicine.

[114] Mashayekhi-Sardoo H., Rezaee R., Karimi G. (2020). An overview of in vivo toxicological profile of thymoquinone. Toxin Rev..

[115] Badary O.A., Al-Shabanah O.A., Nagi M.N., Al-Bekairi A.M., Elmazar M.M.A. (1998). Acute and subchronic toxicity of thymoquinone in mice. Drug Dev. Res..

